# Fibroblasts accelerate islet revascularization and improve long-term graft survival in a mouse model of subcutaneous islet transplantation

**DOI:** 10.1371/journal.pone.0180695

**Published:** 2017-07-03

**Authors:** Marcos Perez-Basterrechea, Manuel Martinez Esteban, Maria Alvarez-Viejo, Tania Fontanil, Santiago Cal, Marta Sanchez Pitiot, Jesus Otero, Alvaro Jesus Obaya

**Affiliations:** 1 Unidad de Trasplantes, Terapia Celular y Medicina Regenerativa, Hospital Universitario Central de Asturias, Oviedo, Spain; 2 Departamento de Biologia Funcional, Universidad de Oviedo, Oviedo, Spain; 3 Departamento de Bioquimica y Biologia Molecular, Universidad de Oviedo, Oviedo, Spain; 4 Unidad de Histopatología Molecular en Modelos Animales de Cáncer, Instituto Universitario Oncológico del Principado de Asturias, Oviedo, Spain; Children's Hospital Boston, UNITED STATES

## Abstract

Pancreatic islet transplantation has been considered for many years a promising therapy for beta-cell replacement in patients with type-1 diabetes despite that long-term clinical results are not as satisfactory. This fact points to the necessity of designing strategies to improve and accelerate islets engraftment, paying special attention to events assuring their revascularization. Fibroblasts constitute a cell population that collaborates on tissue homeostasis, keeping the equilibrium between production and degradation of structural components as well as maintaining the required amount of survival factors. Our group has developed a model for subcutaneous islet transplantation using a plasma-based scaffold containing fibroblasts as accessory cells that allowed achieving glycemic control in diabetic mice. Transplanted tissue engraftment is critical during the first days after transplantation, thus we have gone in depth into the graft-supporting role of fibroblasts during the first ten days after islet transplantation. All mice transplanted with islets embedded in the plasma-based scaffold reversed hyperglycemia, although long-term glycemic control was maintained only in the group transplanted with the fibroblasts-containing scaffold. By gene expression analysis and histology examination during the first days we could conclude that these differences might be explained by overexpression of genes involved in vessel development as well as in β-cell regeneration that were detected when fibroblasts were present in the graft. Furthermore, fibroblasts presence correlated with a faster graft re-vascularization, a higher insulin-positive area and a lower cell death. Therefore, this work underlines the importance of fibroblasts as accessory cells in islet transplantation, and suggests its possible use in other graft-supporting strategies.

## Introduction

Pancreatic islet transplantation has been considered along the last two decades as a promising therapy for beta-cell replacement in patients with type-1 diabetes. This strategy reduce the risks associated to whole-pancreas transplantation and is specially indicated to those patients with unstable glycemic control, hypoglycemia unawareness and/or resistance to intensive insulin-based therapies [1]. Nevertheless, although most of islet recipients remained protected from recurrent hypoglycemia, less than 50% maintained insulin independence five years after islet transplantation [2, 3]. Thus, this long-term loss-of-function evidence the necessity of improving some aspects of islet transplantation, especially those addressing obstacles like engraftment and transplantation site [4].

In addition to a supporting matrix or scaffold, an optimal engraftment site requires access to adequate oxygen and nutrient supplies either from endogenous vasculature or from induced or intrinsic neovascularization [5]. During the first days after transplantation, when the islet graft is avascular, up to 60% of the islet mass can be lost [6]. Thus, graft revascularization plays a critical role in islet viability and function [7], as well as in restoration of the islet-extracellular matrix interactions [8]. Several sites have been proposed as alternative to intrahepatic islet transplantation. Among them, subcutaneous space stands out because of its accessibility and spaciousness, although its poor vascularization constitutes its main limitation [9]. Taking this in mind, improvement of angiogenesis becomes crucial for successful subcutaneous islet transplantation. Several attempts have been conducted, mainly focused on the use of different biomaterials as scaffolds, either alone or in combination with pro-angiogenic factors and, more recently, in combination with accessory cells with graft-supporting properties.

Fibroblasts are a heterogeneous mesenchymal-derived cell population with different characteristics depending on their tissue of origin [10]. These cells collaborate on extracellular matrix and connective tissue formation producing several molecules as metalloproteinases, collagen, fibronectins and proteoglicans [11], which provide support for organ structures, including blood vessels. Moreover, fibroblasts secrete per se or under hypoxic conditions several factors (VEGF, FGF, PDGF, HIF-1α), of which some are potent angiogenic factors [12].

Our group has developed a model for subcutaneous islet transplantation using a plasma-based scaffold containing fibroblasts as graft-supporting accessory cells that allowed long-term glycemic control in diabetic immune-deficient mice [13]. Moreover, combination of fibroblasts with immunomodulatory mesenchymal stem cells (MSCs) was also able to temporarily control blood glucose levels in immune-competent mice [14].

In the present work we have gone in depth using gene expression analysis and immunohistochemistry techniques into the graft-supporting role of fibroblasts in this subcutaneous islet transplantation model. Taking in mind that revascularization begins 2–4 days after transplantation and is mostly complete by 10–14 days [15], we focus our study on this period, when a fast and efficient islet revascularization results critical for an adequate engraftment and long-term function.

## Materials and methods

### Animals

Twelve-week-old male Wistar rats (Animal Facilities of University of Oviedo, Oviedo, Spain) were used as pancreatic islet donors. Six-week-old male Swiss nu/nu mice (Harlan Iberica, Barcelona, Spain) were used as immunodeficient recipients. Mice were rendered diabetic by a single intraperitoneal injection of 225 mg/kg body weight of streptozotocin (Sigma-Aldrich, Madrid, Spain). Diabetes was defined as non-fasting blood glucose (NFBG) levels higher than 450 mg/dL determined in two consecutive days. This study was carried out in strict accordance with the guidelines of the European Union (86/609/EU) and Spanish regulations (BOE 67/8509-12, 1988). Experimental protocols were approved by the Committee for Animal Care and Handling of the University of Oviedo (Permit number: 9-INV-2004). All surgery was performed under Isoflurane anesthesia (IsoFlo^®^, Abbott Laboratories, Abbott Park, IL) and animals were sacrificed using CO_2_. All efforts were made to minimize animal suffering and to reduce the number of animals used.

### Islet graft

#### Plasma

Blood was obtained from Wistar rats by venipuncture into 5-mL sodium citrate-coated sterile tubes (Vacutainer SST^™^ II Advance; Becton-Dickinson, Plymouth, United Kingdom) and centrifuged at 3000 rpm for 15 min. The plasma was collected and frozen until use.

#### Pancreatic islets

As previously described [16], Wistar rat pancreas was distended by an intraductal injection of collagenase type XI (Sigma-Aldrich). The pancreas was then harvested, incubated in a water bath at 37°C for 21 min, and the digested tissue washed in Hank’s balanced salt solution (HBSS, Sigma-Aldrich). Islets were separated by Dextran (Sigma-Aldrich) discontinuous density gradient centrifugation, washed in HBSS, and immediately prepared for transplant. Islet equivalents (IEQs), purity, and viability were calculated by examining dithizone-stained (Sigma-Aldrich) preparations.

#### Skin fibroblasts

As previously described [13], a full-thickness skin biopsy specimen (1 cm^2^) from the right flank of a nu/nu mice was obtained and grinded without previous dermis-epidermis separation. Then, the homogenate was subjected to enzyme digestion with 2 mg/mL collagenase (Sigma-Aldrich) at 37°C for 4 h and the digested tissue suspension was then filtered through a cell strainer (Becton Dickinson) and centrifuged at 1400 rpm for 10 min. The resultant pellet was suspended in Dulbecco’s modified Eagle’s medium (DMEM, Lonza, Basel, Switzerland) supplemented with 10% fetal calf serum (Gibco Invitrogen, Paisley, United Kingdom) and antibiotic-antimycotic solution (50 U/ml penicillin + 50 μg/ml streptomycin, Gibco Invitrogen). Cells were seeded in culture flasks and cultured at 37°C in a humidified atmosphere of 5% CO_2_. Fibroblasts were used after 4–5 passages.

#### Plasma-based scaffold containing islets (PSI)

Fresh pancreatic islets (3000 IEQs) were mixed with 2 mL of plasma alone (PSI) or containing 2 x 10^5^ skin fibroblasts (PSI-F). Then, 5 mg/mL of tranexamid acid (Amchafibrin, Fides-Ecofarm, Barcelona, Spain) was added and the final volume was adjusted to 4 mL with DMEM + 6.75 mM CaCl_2_. The mixture was finally allowed to solidify for 15–20 minutes at 37°C in a humidified atmosphere of 5% CO_2_.

#### Subcutaneous islet transplantation

Transplantation was performed in diabetic mice 7–10 days after STZ injection. The islet graft (PSI or PSI-F) was placed directly over the abdominal musculature after performing a skin incision. NFBG was monitored periodically after transplantation on whole blood samples obtained from the tail vein. Normoglycemia was defined as a NFBG level <200 mg/dL.

### Animal groups

The following groups were established:

Diabetic group (n = 8): Non-transplanted diabetic mice (7–10 days after streptozotocin injection).
Control group (n = 8): Non-transplanted healthy mice.
ISC (n = 8): Diabetic mice subcutaneously transplanted with islets alone.
PSI (n = 16): Diabetic mice subcutaneously transplanted with PSI.
PSI-F (n = 16): Diabetic mice subcutaneously transplanted with PSI-F.

### Intraperitoneal glucose tolerance tests (IPGTTs)

After an overnight fast, the animals received an intraperitoneal glucose bolus (2 g/Kg of body weight) as a 5% solution in Hanks’ Balanced Salt Solution (Lonza), and blood glucose was monitored at 0, 15, 30, 60, 90 and 120 min. To evaluate the response to IPGTTs, the “area under the curve” was calculated for each animal using the trapezoidal rule method [17].

### Subcutaneous islet graft gene expression study

Of each experimental group (PSI and PSI-F), two mice were sacrificed at days 1, 3, 7 and 10 after islet transplantation. Each subcutaneous islet graft was divided in halves: one half was used for total RNA extraction, while the other half was fixed in 4% formaldehyde for histological study.

Whole RNA was isolated using TRIzol (Invitrogen) and purified with the RNeasy Mini Kit (Qiagen). Concentration and quality of samples were determined using a NanoDrop 2000 (Thermoscientific, Wilmington, DE) at A260/A280. Those with the best quality were selected for hybridization with GeneChip^®^ Mouse Genome 430 2.0 array (Affymetrix), following the manufacturer’s instructions. Quality control of microarray data was performed using Affymetrix Expression Console. Data are expressed as base-2 exponents. Array data were deposited at the Gene Expression Omnibus with the accession number GSE84900. Bioinformatic analysis was performed using the Babelomics platform (http://www.babelomics.org).

### Histology

The histological study was performed in subcutaneous grafts of mice sacrificed at days 1, 3, 7, 10 and 90 days after transplantation. After a 24 h fixation in 4% formaldehyde, the specimens were dehydrated in a graded series of ethanol dilutions and finally embedded in paraffin. Sections of 4–6 μm thick were obtained using a microtome HM 350 S (Microm, Waldorf, Germany). Some sections were stained with hematoxylin and eosin (H&E). The presence of functional islets was confirmed by immunohistochemical insulin labelling. Briefly, paraffin sections were deparaffinized and rehydrated in EZ Prep, and placed in Reaction Buffer. The primary antibody (Ab) was polyclonal Guinea pig Anti-Insulin (Dako, Glostrup, Denmark). Sections were incubated at 37°C with primary Ab (1:60) for 1 h in Ab diluent. Some sections (those of 1, 3, 7 and 10 days after transplantation) were incubated with the secondary Ab OmniMap anti-Rb HRP and then, detected with ChromoMap DAB; while other sections (those of 90 days after transplantation) were incubated with a rabbit anti-guinea pig IgG alkaline phosphatase conjugated secondary Ab (Abcam, Cambridge, United Kingdom) and visualized using the alkaline phosphatase-chromogen kit (Fast Red^™^) Ab (Abcam) according to the manufacturer´s instructions. Contrast staining was performed with hematoxylin. The insulin-positive area (expressed in μm^2^) was determined in equivalent areas (6 fields per slide, n = 2 per group at 1, 3, 7 and 10 days or n = 8 per group at 90 days after transplantation). For the insulin-lectin double staining (90 days after transplantation), insulin was performed as previously indicated, although donkey anti-guinea pig Cy5 conjugated secondary Ab (Jackson Laboratories) was used. In addition, *Bandeiraea simplicifolia* FITC-conjugated lectin (Sigma Aldrich) was used to detect vessel, and then, mounted with VECTASHIELD Antifade Mounting Medium with DAPI (Vector Laboratories, Burlington, Canada) to stain nuclei and examined with a laser scanning confocal microscope TCS-SP8X (Leica, Barcelona, Spain). For vessel counting, anti-CD31 polyclonal Ab (Abcam) was used. Sections were incubated at 37°C with primary Ab (1:50) for 32 min. The secondary Ab used was OmniMap anti-Rb HRP and detection was carried out with ChromoMap DAB. Contrast staining was performed with hematoxylin. Vessel number was determined by counting total CD-31 positive vessels observed on the matrix surrounding islets in equivalent areas (6 fields per slide, n = 2 per group at 1, 3, 7 and 10 days or n = 8 per group at 90 days after transplantation). On the other hand, the presence of apoptotic cells was determined by using CF^™^488A TUNEL Assay Apoptosis Detection Kit (Biotium, Hayward, Canada) according to the manufacturer´s instructions. Finally, sections were mounted with VECTASHIELD Antifade Mounting Medium with DAPI (Vector Laboratories) to stain nuclei and examined with a laser scanning confocal microscope. TUNEL(+) cells per area (in mm^2^) was determined in equivalent areas (6 fields per slide, n = 2 per group at 1, 3, 7 and 10 days after transplantation) obtained with the laser scanning confocal microscope. Beta cell proliferation was determined by using a Guinea pig anti-insulin Ab (Abcam) and a rabbit anti-Ki67 Ab (Abcam). Sections were incubated with anti-insulin Ab (1:500) and anti-Ki67 Ab (1:500) for 48h at 4°C. Next, an Anti-rabbit Ig G (Alexa Fluor^®^ 647, Abcam) and an anti-Guinea Pig Ig G (Alexa Fluor^®^ 488, Abcam) were used at 1:1000 and 1:500 respectively. Finally, sections were mounted with VECTASHIELD Antifade Mounting Medium with DAPI (Vector Laboratories) to stain nuclei and examined with a laser scanning confocal microscope. Percentage of Ki67(+) Insulin(+) cells per total Insulin (+) cells were determined in equivalent areas (6 fields per slide, n = 2 per group at 1, 3, 7 and 10 days or n = 8 per group at 90 days after transplantation. All reagents, except where specified, were from Ventana Medical Systems (Roche group, Tucson, AZ).

### Statistical analysis

Results are expressed as mean ± SEM. The significance of differences between two independent groups was calculated using the unpaired Student’s *t*-test. For multiple comparisons, ANOVA tests were conducted followed by the Scheffé *post hoc* test to determine specific differences between groups. A p-value <0.05 was considered statistically significant.

## Results

### Fibroblasts improve long-term survival and function of subcutaneous islet grafts

**Fig 1:**
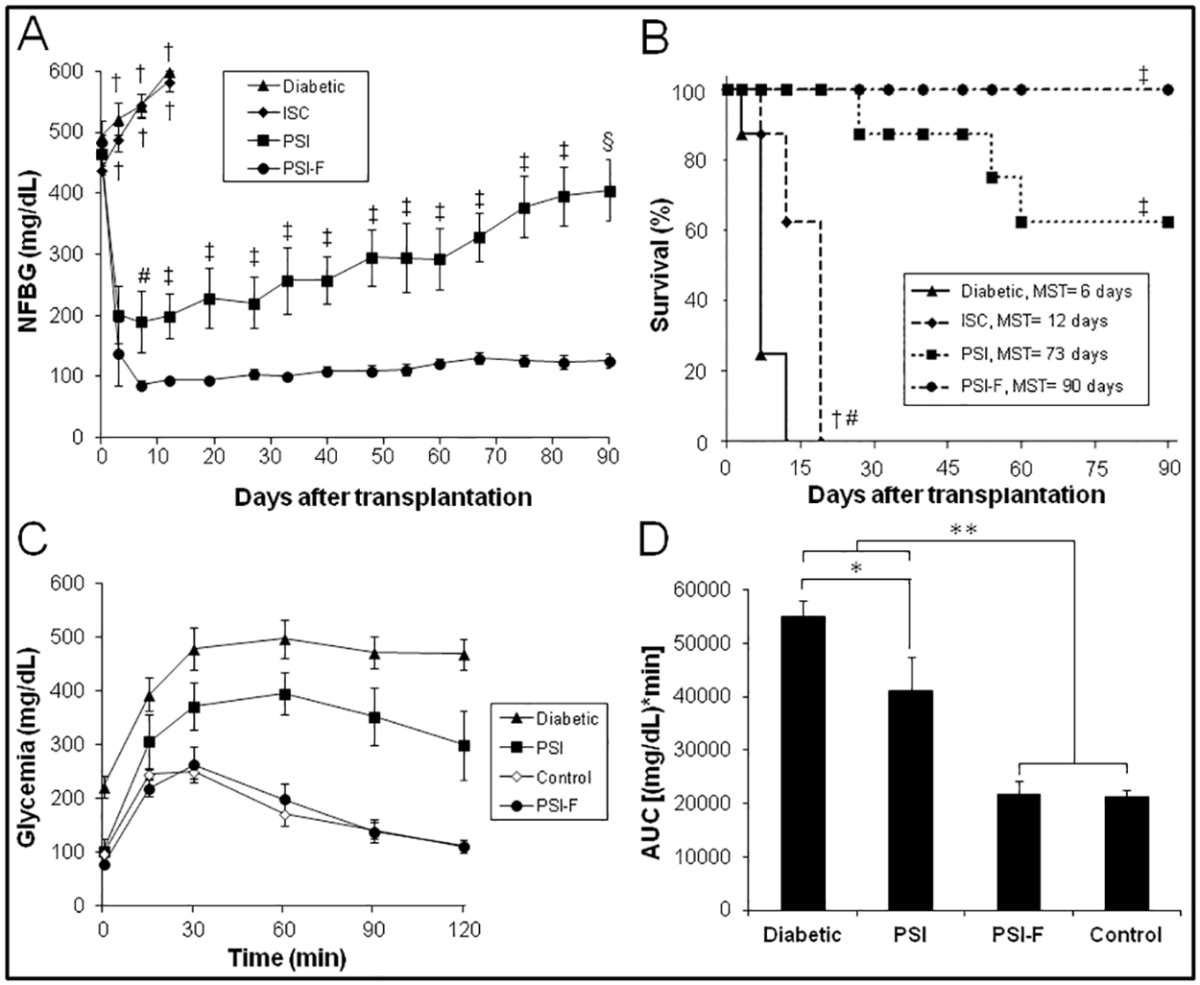
Subcutaneous transplantation of PSI in immunodeficient diabetic mice. (A) Follow up of NFBG in the following groups (n = 8 per group): (▲, Diabetic) non-transplanted diabetic mice; (♦, ISC) diabetic mice subcutaneously transplanted with free islets; (■, PSI) diabetic mice subcutaneously transplanted with PSI; (●, PSI-F) diabetic mice subcutaneously transplanted with PSI containing 2 x 10^5^ fibroblasts. ^†^p<0.001 vs. PSI and PSI-F; ^#^p<0.05 vs. PSI-F; ^‡^p<0.01 vs. PSI-F; ^§^p<0.001 vs. PSI-F. (B) Kaplan-Meier mice survival curve. Groups are the same as detailed in A (n = 8 per group). Mean survival time (MST) is indicated for each group. ^†^p<0.01 vs. Diabetic; ^#^p<0.001 vs. PSI and PSI-F; ^‡^p<0.001 vs. Diabetic. (C) Blood glucose responses to IPGTTs, 90 days after transplantation. Groups are the same as detailed in A, except for (◊, Control) non-transplanted healthy mice (n = 8 per group, except for PSI where n = 5). (D) Area under the curve (AUC) data obtained from IPGTTs. *p<0.05; **p<0.01.

Diabetic nude mice were transplanted subcutaneously with islets embedded into a plasma-based scaffold without cells (PSI) or with fibroblasts (PSI-F). In both groups, hyperglycemia was reversed in the first days after transplantation (Fig 1A). However, significant differences were observed: meanwhile PSI-F mice maintained normoglycemia along the 90 days follow-up, PSI mice showed a progressive deterioration of glycemic control which forced the sacrifice of some animals due to their critical condition (Fig 1B).

On the other hand, when IPGTTs were performed at the end of the 90-days follow-up, clear differences were observed among groups (Fig 1C and 1D). While PSI-F mice showed a good glucose tolerance, similar to that of control mice, PSI mice were not able to reverse hyperglycemia after glucose bolus administration (Fig 1D, 21709±2522 vs. 41004±6341 (mg/dL)*min for PSI-F and PSI, respectively, p<0.01). Nevertheless, PSI mice showed a response significantly better than that showed by non-transplanted diabetic mice (Fig 1D, 41004±6341 vs. 54896±3084 (mg/dL)*min for PSI and Diabetic, respectively, p<0.05).

**Fig 2:**
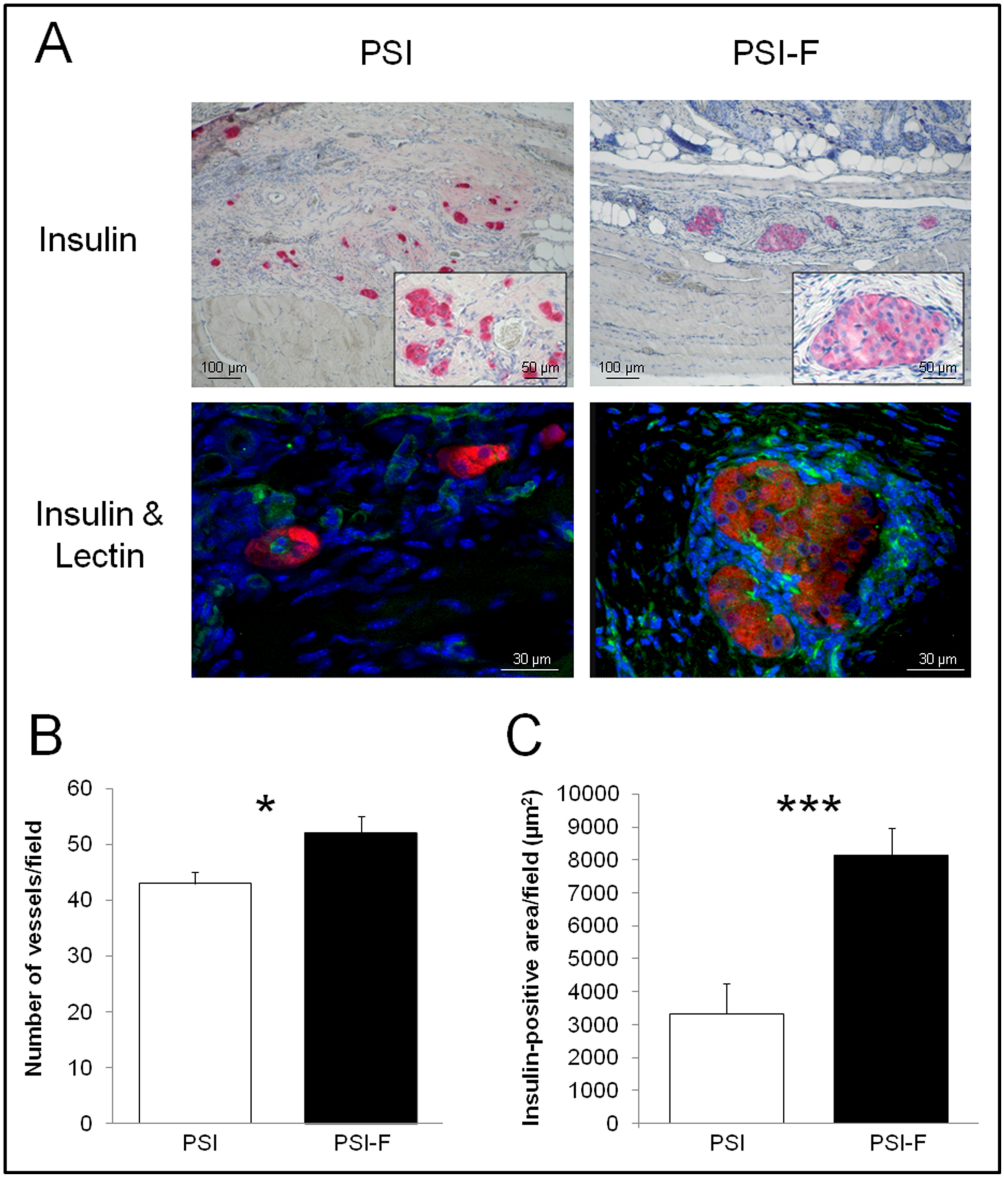
Histology of subcutaneous islet grafts 90 days after transplantation. (A) Insulin immunostaining and insulin/lectin immunofluorescence (insulin in red to detect beta cells and lectin in green to detect vessels) performed on both PSI and PSI-F grafts. (B) Quantification of vessels in the subcutaneous grafts after CD31 immunostaining. (C) Quantification of the insulin-positive area in the subcutaneous grafts. *p<0.05; ***p<0.001.

Regarding to the histological study performed on subcutaneous grafts on day 90 after transplantation, insulin-positive islets and vessels were observed in both PSI and PSI-F mice, although islets in presence of fibroblasts were bigger and showed a better morphology (Fig 2A). Moderate fibrosis was observed in both groups, with islets embedded by a new collagen-like matrix, which substituted the initial plasma-based scaffold. However, this matrix was more organized and vascularized in presence of fibroblasts (Fig 2B, 52±3 vs. 43±2 vessels/field for PSI-F and PSI, respectively, p<0.05), which correlated with the significantly higher insulin area observed in this group (Fig 2C, 8125±836 vs. 3335±917 μm^2^ for PSI-F and PSI, respectively, p<0.001). The increase in insulin area cannot be explained by an induction of beta-cell proliferation in the presence of fibroblasts since a double Ki67/insulin staining showed no differences between both groups (S1 Fig).

### Fibroblasts accelerate subcutaneous islet graft vascularization

Taking in mind the differences found between PSI and PSI-F groups 90 days after transplantation, and the critical influence of the phenomena occurred during the first days after transplantation over long-term results, we focused our study in this early period in order to look for differences among groups. PSI and PSI-F mice were sacrificed at day 1, 3, 7 and 10 after transplantation, and subcutaneous grafts were retrieved and processed both for histological and gene expression analysis.

**Fig 3:**
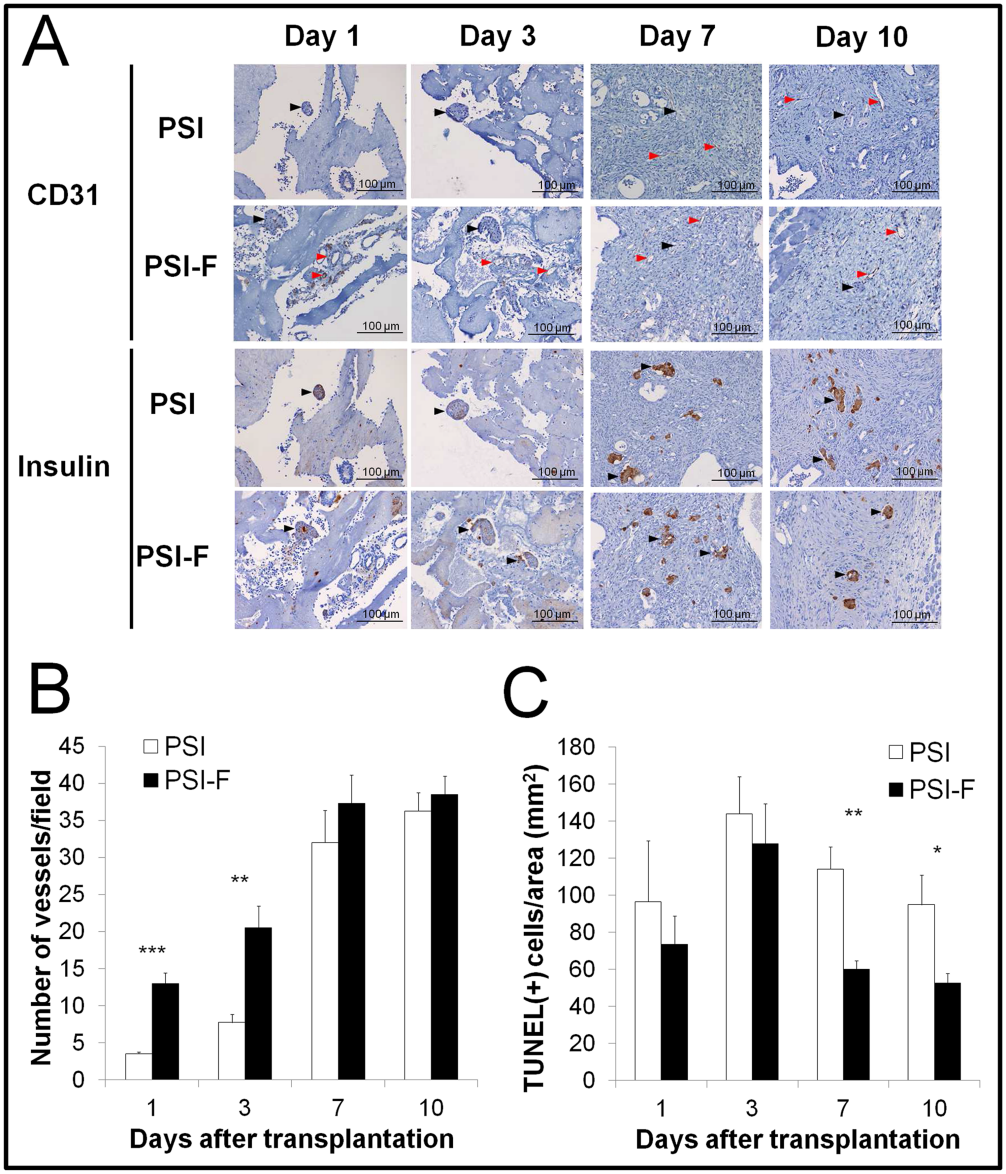
Histology of subcutaneous islet grafts along the first 10 days after transplantation. (A) Histological images of CD31 (to detect vessels) and insulin (to detect beta cells) immunostaining of PSI and PSI-F grafts at days 1, 3, 7 and 10 after transplantation (Black arrows point to islets and red arrows point to vessels). (B) Quantification of vessels in the subcutaneous grafts after CD31 immunostaining at days 1, 3, 7 and 10 after transplantation. (C) Quantification of apoptotic cells per area in PSI and PSI-F grafts at days 1, 3, 7 and 10 after transplantation. *p<0.05; **p<0.01; ***p<0.001.

The histological analysis of the grafts (Fig 3A) showed a faster revascularization in presence of fibroblasts (PSI-F), with a vessel number (Fig 3B) significantly higher at days 1 (3.5±0.3 vs. 13±1.4 vessels/field for PSI and PSI-F, respectively, p<0.001) and 3 (7.75±1.1 vs. 20.5±2.9 vessels/field for PSI and PSI-F, respectively, p<0.01) after transplantation, although these differences diminished afterwards. However, insulin staining began to be notorious on both groups at day 7, with no statistically significant differences between them (Fig 3A). As mentioned before, no significant differences were observed on beta cell proliferation among groups (S1 Fig). Since lack of vascularization during this early period after transplantation can induce cell death, a TUNEL assay was performed. The number of apoptotic cells per field (Fig 3C) was significantly higher in absence of fibroblasts (PSI) on days 7 (114±12 vs. 60±5 TUNEL(+) cells/area for PSI and PSI-F, respectively, p<0.01) and 10 (95±16 vs. 53±5 TUNEL(+) cells/area for PSI and PSI-F, respectively, p<0.05) after transplantation. A fibrotic process was observed in all samples, being the level of fibrosis higher in presence of fibroblasts (days 1 and 3, mild vs. moderate for PSI and PSI-F, respectively; days 7 and 10, moderate vs. severe for PSI and PSI-F, respectively). This fibrotic process seems to be in correlation with the absorption rate of plasma-based scaffold, which seemed to begin earlier in PSI-F mice, where a new-formed collagen-like matrix was observed at day 10 but not in absence of fibroblasts (Fig 3A).

### Fibroblasts exert their activity by inducing a higher expression of pro-angiogenic and pro-survival factors

**Fig 4:**
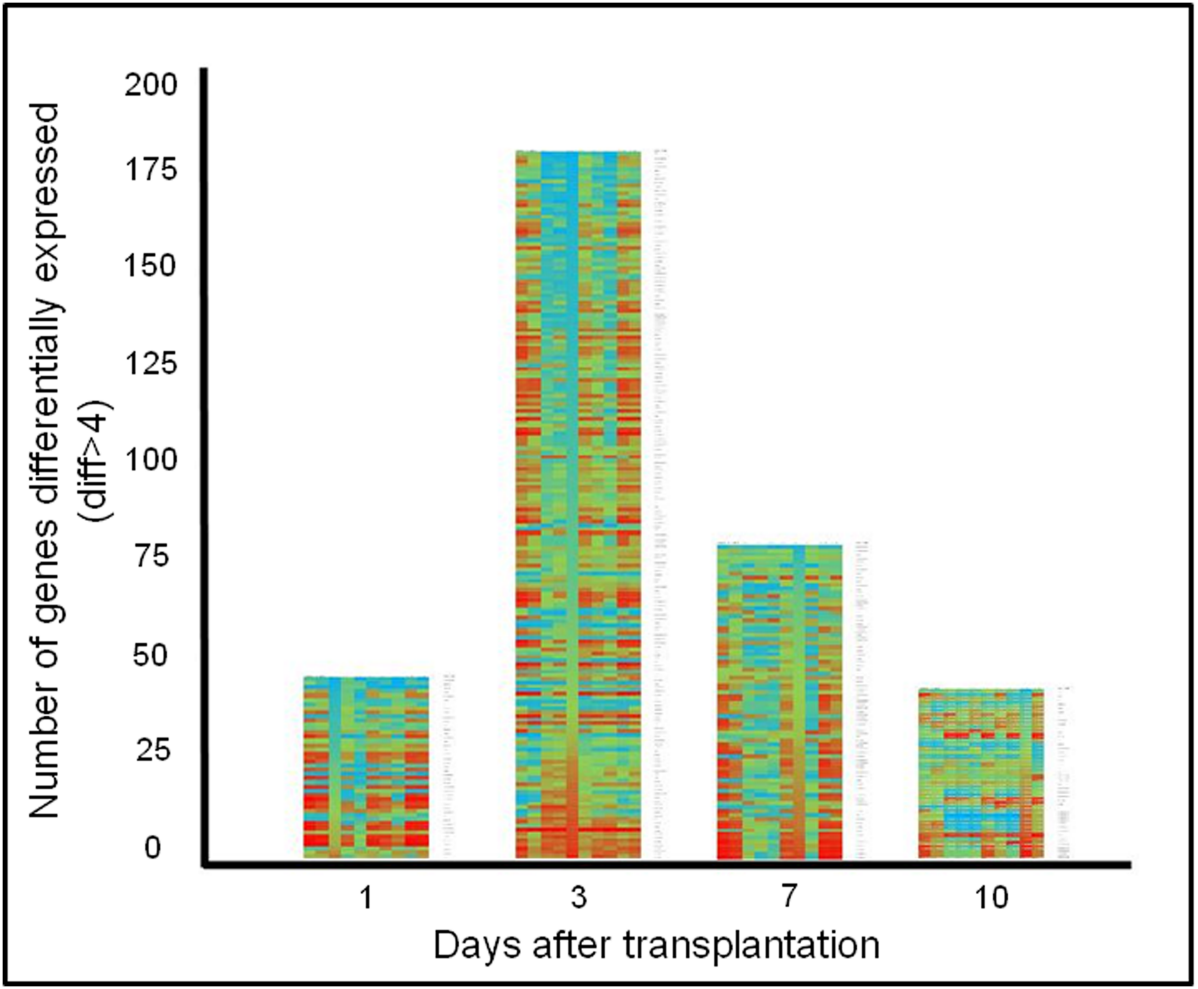
Genes differentially expressed in PSI group vs. PSI-F group. Heat map represents the relative expression levels of identified genes (logFC2 PSI vs. PSI-F) in each day as determined in a GeneChip Mouse Gene 2.0 Array analysis (being green the higher expression and red the lower). Right column indicates gene name. Additionally, in each day the heat map for any individual gene represents the variation of its expression during all the samples taken for our analysis (0, 1, 3, 7 and 10 days). Genes have been arranged from maximum to minimum fold expression differences in any given day after transplantation.

In order to look for putative factors that might be important during islet engraftment and its posterior survival, we performed a gene expression profile of subcutaneous islet grafts. First, we compared profiles between groups with special attention to those differences shown when comparing PSI to PSI-F RNA profiles. In this sense, we started our analysis by looking at differences of 4-fold difference in gene expression (logFC2) within the day after islet transplantation and between samples from PSI-F and PSI (Fig 4).

**Fig 5:**
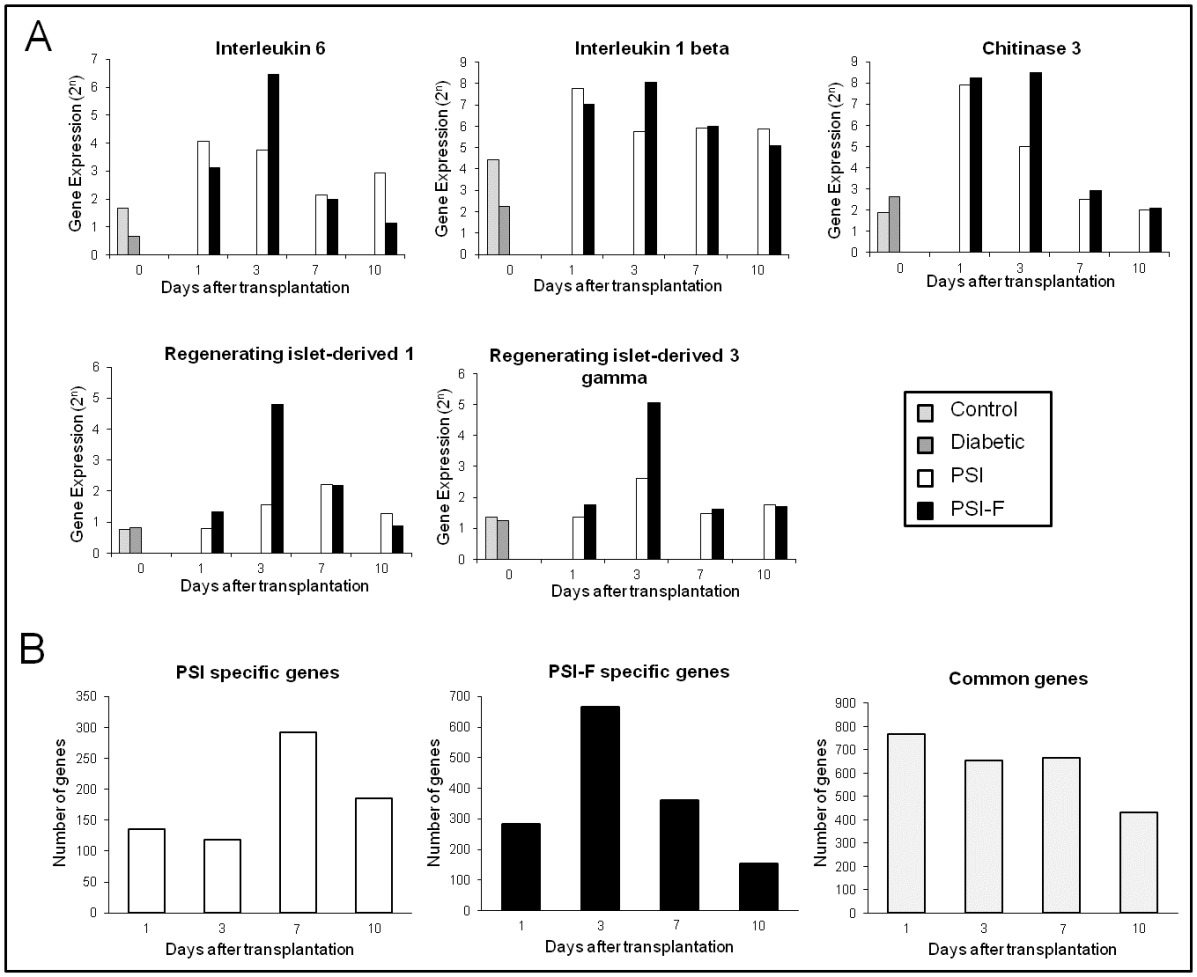
Genes expressed on the first ten days after transplantation on subcutaneous islet grafts. (A) Pattern of expression of Interleukin 6, Interleukin 1 beta, Chitinase 3, Regenerating islet-derived 1 and Regenerating islet-derived 3 gamma after transplantation. (B) Number of genes specifically expressed in PSI, PSI-F and commonly expressed in both groups when compared with Diabetic group.

The maximum difference between both groups occurred at day 3 after islet transplantation, with 177 genes differentially expressed. Day 7 was the following day in terms of number of genes differentially expressed (78 genes), followed by day 1 (44 genes) and day 10 (39 genes). When we analyzed these genes using bioinformatic tools available at www.babylomics.com we could not observe important affected pathways although important genes involved in inflammation, vascularization and islet regeneration and survival could be detected such as Interleukin-6 (IL-6), Interleukin-1beta (IL-1β), Chitinase-3 (Chi-3), Regenerating islet-derived 1 (REG-1) and Regenerating islet-derived 3 gamma (REG-3gamma) (Fig 5A). In all these genes, an overexpression in presence of fibroblasts could be observed at day 3.

Since all mice were diabetic before islet transplantation, we tried to analyze our array data with a different approach. In this sense we compared each transplanted group (PSI and PSI-F) with the Diabetic group. Then, we compared the different expressed genes in search for genes specifically present in the PSI vs. Diabetic, those specifically present in the PSI-F vs. Diabetic, and those common to both groups (Fig 5B) (S1–S4 Tables). In this type of analysis we could also observe that in the first three comparisons (days 1, 3 and 7 after transplantation) more genes were specific for the PSI-F group (281, 666 and 360 genes, respectively). At these days, the genes specifically expressed in the PSI group were 135, 118 and 292 genes, respectively. As opposed, more genes were specific for the PSI group than for the PSI-F group on day 10 (185 vs. 153 genes, for PSI and PSI-F, respectively). Regarding common genes expressed in both groups the numbers were 768, 665, 667 and 433 genes for days 1, 3, 7 and 10 (Fig 5B). Since long term survival of islets after transplantation is achieved only in the PSI-F group we used the specifically expressed genes in this group in search of pathways in which they were involved using the babylomics platform (Table 1). In this type of analysis we were able to observe that these genes can be grouped in important pathways that could explain long-term survival of transplanted islets. Thus, important pathways on vascularization were represented by genes expressed specifically in PSI-F samples. It is noteworthy how pathways such as “blood vessel development”, “vasculature development”, “blood vessel morphogenesis” or “response to hypoxia” showed up on day 3; and pathways related to cell death appeared on day 7 (Table 1).

**Table 1:** Pathways represented by specifically expressed genes in PSI-F (www.babelomics.org).

**Day 1**		
**Pathway**	**#term**	**adj pvalue**
Cytokine-cytokine receptor interaction—Mus musculus (mouse)	mmu04060	7.82594E-3
Toll-like receptor signaling pathway—Mus musculus (mouse)	mmu04620	4.21797E-3
Cytosolic DNA-sensing pathway—Mus musculus (mouse)	mmu04623	2.37377E-2
immune response	GO:0006955	1.1332E-5
response to fungus	GO:0009620	1.83454E-2
positive regulation of B cell activation	GO:0050871	2.26813E-2
tissue morphogenesis	GO:0048729	2.97847E-2
cell activation	GO:0001775	2.97847E-2
regulation of immune system process	GO:0002682	2.97847E-2
mononuclear cell proliferation	GO:0032943	2.97847E-2
lymphocyte proliferation	GO:0046651	2.97847E-2
cellular response to stimulus	GO:0051716	4.81378E-2
tissue development	GO:0009888	4.81378E-2
**Day 3**		
**Pathway**	**#term**	**adj pvalue**
Leukocyte transendothelial migration—Mus musculus (mouse)	mmu04670	2.37835E-2
Insulin signaling pathway—Mus musculus (mouse)	mmu04910	3.15866E-2
Hematopoietic cell lineage—Mus musculus (mouse)	mmu04640	3.15866E-2
enzyme linked receptor protein signaling pathway	GO:0007167	4.10724E-8
cell migration	GO:0016477	2.20784E-7
cellular component movement	GO:0006928	5.94421E-7
organ morphogenesis	GO:0009887	1.28216E-5
transmembrane receptor protein tyrosine kinase signaling pathway	GO:0007169	2.33507E-5
blood vessel development	GO:0001568	8.83843E-5
vasculature development	GO:0001944	1.01758E-4
regulation of cell communication	GO:0010646	1.39045E-4
blood vessel morphogenesis	GO:0048514	1.35708E-3
response to hypoxia	GO:0001666	2.5248E-3
**Day 7**		
**Pathway**	**#term**	**adj pvalue**
Asthma—Mus musculus (mouse)	mmu05310	3.49504E-4
Systemic lupus erythematosus—Mus musculus (mouse)	mmu05322	5.06548E-4
Allograft rejection—Mus musculus (mouse)	mmu05330	5.06548E-4
Cell adhesion molecules (CAMs)—Mus musculus (mouse)	mmu04514	5.06548E-4
Autoimmune thyroid disease—Mus musculus (mouse)	mmu05320	1.52164E-3
Primary immunodeficiency—Mus musculus (mouse)	mmu05340	2.95021E-3
Chemokine signaling pathway—Mus musculus (mouse)	mmu04062	2.95021E-3
Cytosolic DNA-sensing pathway—Mus musculus (mouse)	mmu04623	3.49209E-3
Leukocyte transendothelial migration—Mus musculus (mouse)	mmu04670	8.23078E-3
Graft-versus-host disease—Mus musculus (mouse)	mmu05332	1.38583E-2
Apoptosis	GO:0006915	2.03641E-2
response to oxidative stress	GO:0006979	8.94815E-3
cell death	GO:0008219	2.03641E-2
cell proliferation	GO:0008283	2.9882E-3
programmed cell death	GO:0012501	2.29903E-2
regulation of cell proliferation	GO:0042127	3.59673E-3
defense response	GO:0006952	3.75767E-5
response to other organism	GO:0051707	8.94815E-3
leukocyte mediated immunity	GO:0002443	1.11433E-3
immune response	GO:0006955	7.64244E-13
**Day 10**		
**Pathway**	**#term**	**adj pvalue**
Hematopoietic cell lineage—Mus musculus (mouse)	mmu04640	4.53859E-2
Asthma—Mus musculus (mouse)	mmu05310	4.89735E-2
antigen processing and presentation of exogenous antigen	GO:0019884	3.04511E-3

Significant _kegg_0.05 and significant_go_biological_process_3_9_0.05

## Discussion

In the present work, we have observed that the use of a plasma-based scaffold alone (PSI) improved islet engraftment, reversed hyperglycemia and achieved short-term glycemic control, although long-term control was only achieved when fibroblasts were included in the scaffold before transplantation (PSI-F). The gene expression study performed on the first days after transplantation showed how genes related with angiogenesis and β-cell regeneration were over-expressed in presence of fibroblasts, which correlated with the faster islet graft revascularization, the lower islet lost and the better glycemic control observed in the transplanted animals of this group.

It is known that plasma-derived scaffold provide to the transplanted islets the initial mechanical support necessary for graft survival and function [8]. Fibrin is a natural biomaterial which has been used previously to promote engraftment mainly at extrahepatic sites [18–20]. While those fibrin hydrogels are usually obtained by combination of commercially available fibrin with thrombin, our plasma-based scaffold is obtained by mixing blood plasma with calcium, avoiding the use of fibrinogen or any exogenous pro-coagulant agent. Thus, this plasma-derived scaffold constitutes a cheaper and safer fibrin-containing hydrogel. In our work, transplantation of islets combined with the plasma-based scaffold alone (PSI group) reversed hyperglycemia, although was unable to maintain long-term glycemic control. These results indicate the necessity of complementing the effect of this scaffold by an additional supporting therapy.

Since islet graft is avascular after isolation, its survival would depend on oxygen and nutrient diffusion from the surrounding microenvironment during the first days after transplantation. Therefore, a quick reestablishment of a functional vasculature is essential for its immediate engraftment and thus, for long-term islet survival and function. In spite of the several “pros” of the subcutaneous space, its poor vascularization becomes its main “con” as alternative to the portal vein infusion. Several factor-based strategies have been attempted in order to overcome this, although most of them showed limited efficacy [21–27]. Incorporation of accessory cells with islet-supporting properties could provide several of these factors with precise control over timing, dose delivery and effect duration [28]. In our model, fibroblasts were incorporated to the plasma-derived scaffold as graft-supporting cells (PSI-F group). The higher amount of vessels and the lower number of apoptotic cells found in PSI-F grafts during the first days after transplantation suggest that fibroblasts promoted early islet revascularization. Although fibrosis usually constitutes a pathologic and undesired phenomenon in tissue regeneration, the fibrotic process observed in this model allowed creating a new vascularized ECM at the subcutaneous space which is home to the transplanted pancreatic islets. The presence of fibroblasts in the graft enhanced this fibrotic process and increased vascularization, leading to a higher organized and vascularized ECM that, improved islet engraftment which finally allowed long-term graft survival and function in this group of mice.

The gene expression analysis performed on the islet grafts during the first days after transplantation revealed over-expression of IL-6, IL1-β and Chi-3 in the group transplanted with fibroblasts. It is well known that most of these cytokines have a great pro-angiogenic activity [29]. IL-6 has been previously related with revascularization, function and survival of islets when co-cultured with MSCs [30], while IL-1β has been identified as the principal effector of Myc-induced islet angiogenesis [31]. On the other hand, Chi-3 is a lectin that has been also related with angiogenesis, although mainly associated with cancer progression and metastasis [32, 33]. Additionally to their role in islet revascularization, both IL-6 and IL-1β seem to collaborate on the up-regulation of GLP-1 and insulin production in pancreatic islets [34, 35]. On the other hand, REG-1 and REG-3gamma were also over-expressed in presence of fibroblasts during this early post-transplant period. REG family comprises several lectin-like proteins that have been related with pancreatic β-cell regeneration [36–39]. Furthermore, some studies have shown that some cytokines, as IL-6 and IL-22, can activate the expression of these REG proteins [40–43]. Taking this in mind, REG proteins could be related with the β-cell proliferation induced by several cytokines as IL-6 [44]. In our work, overexpression of cytokines and REG proteins in presence of fibroblasts correlated with the faster islet re-vascularization and the lower level of apoptosis observed, favouring long-term islet survival and function. Recently, an interesting *in vitro* study showed how dermal fibroblasts can promote islet survival by secretion of several cytokines and extracellular matrix proteins [45]. Therefore, our results corroborate those findings in an “in vivo” model, highlighting the possible use of fibroblasts as graft-supporting cells, not only in islet transplantation, but also in those other transplants where a fast graft re-vascularization could be needed. No significant differences were observed in the expression of pro-angiogenic, hypoxia or apoptosis-related genes. In supplementary figure 2 (S2 Fig) we show how the kinetics and patterns of expression for several genes involved in islet survival or implicated in islet death as described by Velmurugan et al [46] were very similar between both groups (PSI-F and PSI). No detection of individual known genes involved in these pathways could also be attributable to the threshold we established in the gene selection process, where a minimum 4-fold difference in gene expression between groups was required to be considered as significant. However, if 1- to 3-fold difference were accepted, an increase in the expression of some pro-angiogenic genes (VEGF A, VEGF C, Angiopoietin-4, FGF-4, FGF-12, PDGF-1 and PDGF-B), as well as of the anti-apoptotic gen BCL-2, was observed in presence of fibroblasts. In fact, it is possible that not only an individual or a small group of genes or factors are implicated in engraftment, islet survival, revascularization and even a proper link to nerve terminals but rather larger groups controlling whole pathways of tissue morphogenesis are implicated. In this sense, by bioinformatic analysis of our arrays, we have been able to observe how the presence of fibroblast is able to affect such pathways.

Tissue engineering approaches constitute a promising alternative in order to improve engraftment in islet transplantation. In addition to the scaffold, the election of the cell type becomes of the utmost importance. In this work, we have shown how the use of fibroblasts as graft-supporting cells promoted the expression of genes related with angiogenesis and β-cell regeneration, decreasing the lost of islets associated to the first days after transplantation and thus, favoring long-term islet survival and function. Since both plasma and dermal fibroblasts are of autologous origin and can be easily obtained; this model constitutes a biocompatible, cheap, safe and reproducible tissue engineering approach available to be used in the clinic. However, further work is required in order to clarify the specific secretome of fibroblasts in this model, allowing their possible use in other graft-supporting strategies. Furthermore, combination of this islet transplantation model with new immunoregulatory strategies [47] as the use of immunomodulatory cells, such as MSCs [14, 48], dendritic cells [49], hematopoietic stem cells [50] or cord blood-derived stem cells [51], as well as other tolerogenic strategies [52], would be necessary in order to achieve long-term survival of islet allo- or xenografts in immunocompetent recipients, avoiding or reducing the use of immunosuppressive drugs and, perhaps, achieving reversal of diabetes.

## Supporting information

S1 Fig Beta cell proliferation in subcutaneous islet grafts at 3 and 90 days after transplantation. A) Immunohistochemical staining of insulin (green), nuclear proliferating marker ki67 (red) and nucleus (in blue). B) Quantification of proliferating beta cells.
(TIF)

S2 Fig Kinetics of fold changes in expression of genes involved in islet survival or islet death. (TIF)

S1 Table Genes specifically or commonly expressed at day 1 after transplantation. (PDF)

S2 Table Genes specifically or commonly expressed at day 3 after transplantation. (PDF)

S3 Table Genes specifically or commonly expressed at day 7 after transplantation. (PDF)

S4 Table Genes specifically or commonly expressed at day 10 after transplantation. (PDF)
