# Supplementary figures and images for: Fibroblasts accelerate islet revascularization and improve long-term graft survival in a mouse model of subcutaneous islet transplantation
Source: PLoS One. 2017 Jul 3;12(7):e0180695. doi: 10.1371/journal.pone.0180695 (PMC5495486; doi:10.1371/journal.pone.0180695)

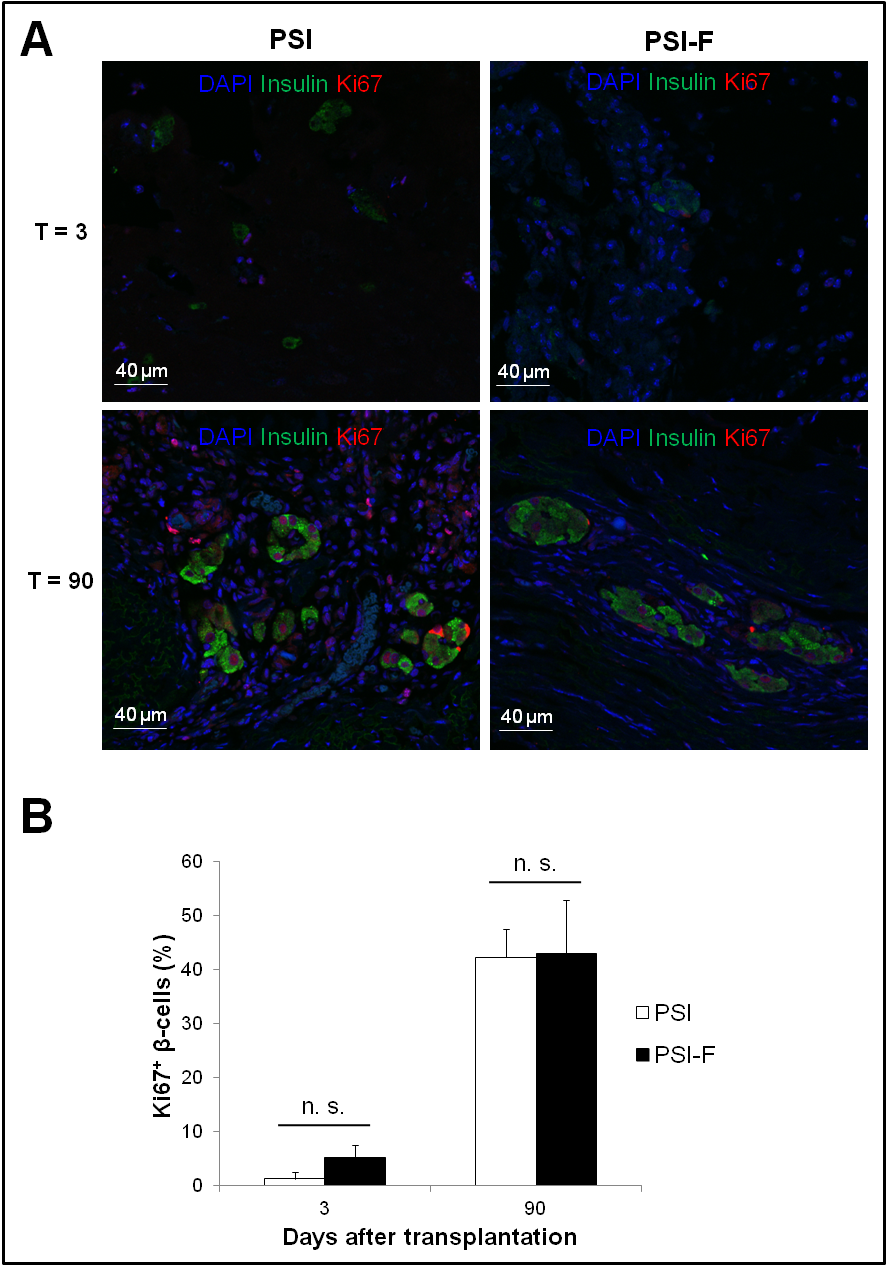

Supplement: S1 Fig — A) Immunohistochemical staining of insulin (green), nuclear proliferating marker ki67 (red) and nucleus (in blue). B) Quantification of proliferating beta cells. (TIF) [file pone.0180695.s001.tif]

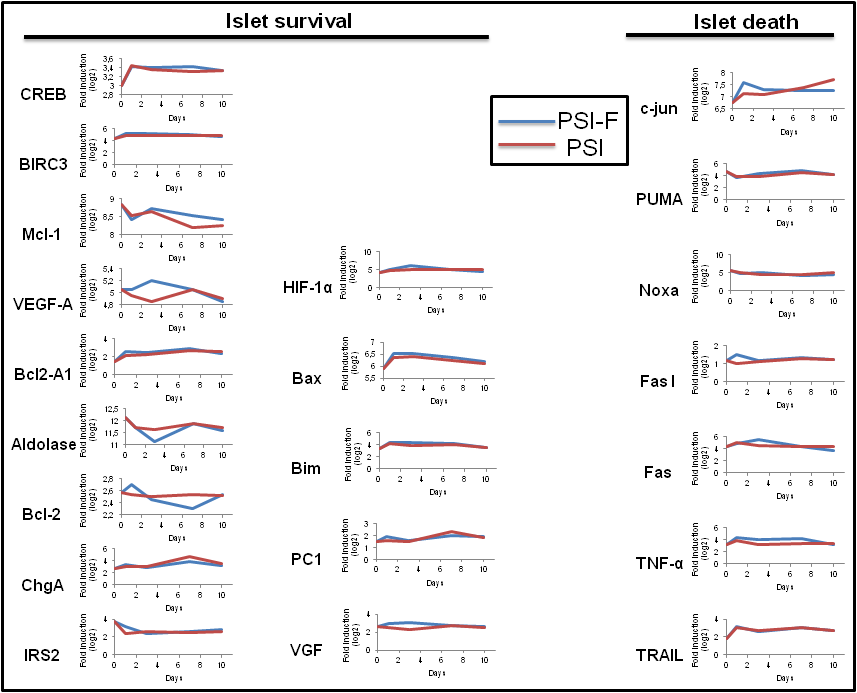

Supplement: S2 Fig — (TIF) [file pone.0180695.s002.tif]
