# Supplementary material for: Fibroblasts accelerate islet revascularization and improve long-term graft survival in a mouse model of subcutaneous islet transplantation
Source: PLoS One. 2017 Jul 3;12(7):e0180695. doi: 10.1371/journal.pone.0180695 (PMC5495486; doi:10.1371/journal.pone.0180695)
Supplement: S1 Table — (PDF) [file pone.0180695.s003.pdf]

Day 1

Prob SET_ID

Specific PSI-F genes	Specific PSI genes	Common genes
1415861_at	1416152_a_at	1415712_at
1416002_x_at	1416505_at	1415983_at
1416332_at	1416687_at	1416053_at
1416380_at	1417262_at	1416151_at
1416407_at	1417263_at	1416191_at
1416530_a_at	1417697_at	1416200_at
1416627_at	1417954_at	1416226_at
1416832_at	1418344_at	1416246_a_at
1417101_at	1418534_at	1416295_a_at
1417155_at	1418626_a_at	1416296_at
1417234_at	1419075_s_at	1416318_at
1417256_at	1419100_at	1416333_at
1417580_s_at	1419219_at	1416356_at
1417717_a_at	1419449_a_at	1416431_at
1417765_a_at	1420249_s_at	1416527_at
1417870_x_at	1420398_at	1416561_at
1418028_at	1421186_at	1416576_at
1418061_at	1421854_at	1416617_at
1418126_at	1422672_at	1416714_at
1418133_at	1422868_s_at	1416776_at
1418173_at	1423233_at	1416871_at
1418317_at	1423396_at	1416897_at
1418345_at	1423723_s_at	1416926_at
1418396_at	1424157_at	1416935_at
1418440_at	1424271_at	1416981_at
1418487_at	1424296_at	1416983_s_at
1418652_at	1424443_at	1416985_at
1418825_at	1424495_a_at	1417025_at
1418946_at	1424717_at	1417104_at
1419060_at	1425040_at	1417141_at

1419128_at
1419132_at
1419135_at
1419149_at
1419369_at
1419378_a_at
1419404_s_at
1419455_at
1419465_at
1419466_at
1419555_at
1419676_at
1419697_at
1420161_at
1420465_s_at
1420657_at
1420991_at
1420992_at
1421106_at
1421578_at
1421680_at
1421998_at
1422095_a_at
1422523_at
1422601_at
1422633_at
1422783_a_at
1422808_s_at
1422962_a_at
1423100_at
1423174_a_at
1423175_s_at
1423182_at
1423294_at
1423375_at
1425431_at
1425480_at
1425655_at
1425958_at
1426536_at
1426958_at
1427180_at
1427256_at
1427544_a_at
1427584_at
1427884_at
1428129_at
1428306_at
1428748_at
1428768_at
1429297_at
1429478_at
1429679_at
1429693_at
1430485_at
1430991_at
1431830_at
1432205_a_at
1433466_at
1433902_at
1434068_s_at
1434362_at
1434430_s_at
1434618_at
1434940_x_at
1435059_at
1435190_at
1435271_at
1435426_s_at
1435585_at
1417244_a_at
1417266_at
1417268_at
1417273_at
1417292_at
1417314_at
1417392_a_at
1417426_at
1417522_at
1417523_at
1417620_at
1417676_a_at
1417793_at
1417802_at
1417803_at
1417813_at
1417821_at
1417876_at
1417936_at
1417961_a_at
1418008_at
1418099_at
1418131_at
1418174_at
1418188_a_at
1418189_s_at
1418191_at
1418204_s_at
1418206_at
1418240_at
1418248_at
1418261_at
1418293_at
1418323_at
1418340_at

1423428_at	1435639_at	1418379_s_at
1423467_at	1436472_at	1418392_a_at
1423570_at	1436594_at	1418465_at
1423754_at	1437121_at	1418480_at
1424338_at	1437240_at	1418483_a_at
1424475_at	1437245_at	1418580_at
1424518_at	1437576_at	1418612_at
1424526_a_at	1437904_at	1418641_at
1424654_at	1438104_at	1418666_at
1424824_at	1438130_at	1418697_at
1424832_at	1438349_at	1418706_at
1424923_at	1438377_x_at	1418742_at
1425228_a_at	1438483_at	1418747_at
1425303_at	1438676_at	1418806_at
1425394_at	1438796_at	1418809_at
1425405_a_at	1439764_s_at	1418826_at
1425567_a_at	1441233_at	1418842_at
1425663_at	1441956_s_at	1418930_at
1425719_a_at	1441958_s_at	1418932_at
1425801_x_at	1442222_at	1418936_at
1425829_a_at	1442538_at	1418945_at
1426276_at	1442798_x_at	1418989_at
1426383_at	1443323_at	1419004_s_at
1426487_a_at	1443540_at	1419042_at
1426726_at	1443621_at	1419043_a_at
1426852_x_at	1444179_at	1419098_at
1426871_at	1444728_at	1419099_x_at
1426970_a_at	1446006_at	1419120_at
1427007_at	1447494_at	1419186_a_at
1427055_at	1447830_s_at	1419194_s_at
1427280_at	1448124_at	1419209_at
1427378_at	1448239_at	1419249_at
1427511_at	1448995_at	1419282_at
1428186_at	1449282_at	1419296_at
1428273_at	1449310_at	1419309_at

1428352_at
1428392_at
1428485_at
1428660_s_at
1428767_at
1428838_a_at
1428853_at
1429006_s_at
1429053_at
1429247_at
1429413_at
1429469_at
1429530_a_at
1429598_at
1430036_at
1430460_at
1430575_a_at
1430576_at
1431339_a_at
1431805_a_at
1433596_at
1434046_at
1434275_at
1434372_at
1434413_at
1434628_a_at
1434777_at
1435077_at
1435110_at
1435125_at
1435313_at
1435562_at
1435872_at
1435903_at
1435945_a_at
1449341_a_at
1450714_at
1451053_a_at
1451289_at
1451319_at
1451762_a_at
1451989_a_at
1452049_at
1452093_at
1452374_at
1452610_at
1452857_at
1452881_at
1453590_at
1453775_at
1454240_at
1454768_at
1454967_at
1455096_at
1455778_at
1455967_at
1456288_at
1456632_at
1456854_at
1456864_at
1456956_at
1457532_at
1457721_at
1457970_at
1458099_at
1459202_at
1459948_at
1460121_at
1460521_a_at
1460600_at
1419321_at
1419323_at
1419329_at
1419394_s_at
1419431_at
1419463_at
1419474_a_at
1419480_at
1419481_at
1419482_at
1419483_at
1419487_at
1419507_at
1419526_at
1419537_at
1419549_at
1419561_at
1419569_a_at
1419573_a_at
1419591_at
1419598_at
1419599_s_at
1419603_at
1419604_at
1419609_at
1419610_at
1419627_s_at
1419631_at
1419707_at
1419714_at
1419728_at
1419764_at
1419767_at
1419840_at
1419874_x_at

1436032_at
1436037_at
1436236_x_at
1436309_at
1436397_at
1436398_at
1436453_at
1436515_at
1436532_at
1436557_at
1436562_at
1436576_at
1436613_at
1436737_a_at
1436791_at
1436913_at
1437162_at
1437303_at
1437445_at
1437558_at
1437668_at
1437751_at
1437788_at
1438059_at
1438259_at
1438306_at
1438345_at
1438385_s_at
1438403_s_at
1438475_at
1438487_s_at
1438531_at
1438814_at
1438879_at
1439163_at

1420310_at
1420330_at
1420331_at
1420358_at
1420361_at
1420380_at
1420394_s_at
1420409_at
1420415_at
1420438_at
1420464_s_at
1420498_a_at
1420538_at
1420575_at
1420599_at
1420671_x_at
1420674_at
1420686_at
1420697_at
1420699_at
1420703_at
1420728_at
1420751_at
1420779_at
1420804_s_at
1420915_at
1420970_at
1421008_at
1421009_at
1421074_at
1421075_s_at
1421187_at
1421188_at
1421217_a_at
1421228_at

1439221_s_at
1439638_at
1439773_at
1439831_at
1440010_at
1440068_at
1440123_at
1440347_at
1440720_s_at
1440789_at
1440799_s_at
1440840_at
1440883_at
1441083_at
1441111_at
1441231_at
1441315_s_at
1441346_at
1441991_at
1442044_at
1442169_at
1442358_at
1442416_at
1442426_at
1442640_at
1443167_at
1443299_at
1443591_at
1443858_at
1444061_at
1444447_at
1444494_at
1444740_at
1445866_at
1445873_at

1421262_at
1421326_at
1421408_at
1421457_a_at
1421492_at
1421525_a_at
1421551_s_at
1421589_at
1421596_s_at
1421644_at
1421689_at
1421691_at
1421694_a_at
1421698_a_at
1421856_at
1421911_at
1421977_at
1422013_at
1422041_at
1422046_at
1422062_at
1422124_a_at
1422160_at
1422190_at
1422191_at
1422209_s_at
1422264_s_at
1422317_a_at
1422324_a_at
1422341_s_at
1422430_at
1422446_x_at
1422447_at
1422562_at
1422570_at

1446504_at
1446511_at
1446553_at
1446649_at
1447116_at
1447211_at
1448163_at
1448377_at
1448380_at
1448421_s_at
1448452_at
1448457_at
1448470_at
1448485_at
1448601_s_at
1448823_at
1448932_at
1449161_at
1449378_at
1449533_at
1449873_at
1449901_a_at
1450032_at
1450264_a_at
1450475_at
1450495_a_at
1450641_at
1450672_a_at
1450699_at
1450753_at
1451097_at
1451374_x_at
1451415_at
1451478_at
1451626_x_at

1422628_at
1422755_at
1422771_at
1422875_at
1422903_at
1422932_a_at
1422953_at
1422973_a_at
1422977_at
1422978_at
1423017_a_at
1423150_at
1423547_at
1423555_a_at
1423569_at
1423590_at
1423753_at
1423768_at
1423858_a_at
1423860_at
1424033_at
1424211_at
1424254_at
1424302_at
1424312_at
1424339_at
1424349_a_at
1424375_s_at
1424524_at
1424552_at
1424655_at
1424683_at
1424713_at
1424727_at
1424737_at

1451755_a_at
1451821_a_at
1452067_at
1452178_at
1452287_at
1452345_at
1452483_a_at
1452500_at
1452606_at
1453009_at
1453136_at
1453299_a_at
1453304_s_at
1453344_at
1453455_at
1453757_at
1454734_at
1454806_at
1455007_s_at
1455136_at
1455220_at
1455401_at
1455787_x_at
1455886_at
1456156_at
1456395_at
1456688_at
1456705_at
1457321_at
1457586_at
1457742_at
1457779_at
1458018_at
1458065_at
1458382_a_at

1424754_at
1424775_at
1424857_a_at
1424921_at
1424927_at
1424965_at
1425001_at
1425025_at
1425065_at
1425099_a_at
1425214_at
1425225_at
1425237_at
1425374_at
1425407_s_at
1425420_s_at
1425430_at
1425434_a_at
1425435_at
1425451_s_at
1425548_a_at
1425598_a_at
1425609_at
1425662_at
1425850_a_at
1425860_x_at
1425863_a_at
1425872_at
1425894_at
1425917_at
1425951_a_at
1426039_a_at
1426203_at
1426278_at
1426413_at

1458603_at
1459170_at
1460048_at
1460231_at
1460463_at
AFFX-b-ActinMur/M12481_M_at

1426415_a_at
1426454_at
1426505_at
1426604_at
1426774_at
1426806_at
1426851_a_at
1426971_at
1427041_at
1427076_at
1427102_at
1427115_at
1427118_at
1427179_at
1427200_at
1427211_at
1427290_at
1427301_at
1427321_s_at
1427327_at
1427339_at
1427365_at
1427366_at
1427381_at
1427388_at
1427397_at
1427483_at
1427540_at
1427549_s_at
1427556_at
1427566_at
1427719_s_at
1427747_a_at
1427751_a_at
1427892_at

1427994_at
1428007_at
1428018_a_at
1428079_at
1428083_at
1428130_at
1428288_at
1428294_at
1428492_at
1428786_at
1428787_at
1428926_at
1429140_at
1429169_at
1429184_at
1429235_at
1429524_at
1429525_s_at
1429570_at
1429775_a_at
1429831_at
1429914_at
1429944_at
1429947_a_at
1429954_at
1429957_at
1430126_at
1430132_at
1430447_a_at
1430509_at
1430579_at
1430581_at
1430584_s_at
1430612_at
1430623_s_at

1430635_at
1430655_at
1430669_at
1430700_a_at
1430703_at
1430731_at
1431166_at
1431171_at
1431182_at
1431504_at
1431591_s_at
1431650_at
1431705_a_at
1431836_x_at
1431843_a_at
1432026_a_at
1432540_at
1432548_at
1432885_at
1433147_at
1433434_at
1433465_a_at
1433593_at
1433617_s_at
1433678_at
1433711_s_at
1433741_at
1433836_a_at
1433837_at
1433877_at
1433963_a_at
1434067_at
1434099_at
1434100_x_at
1434129_s_at

1434139_at
1434202_a_at
1434350_at
1434380_at
1434425_at
1434438_at
1434457_at
1434955_at
1434980_at
1435144_at
1435263_at
1435264_at
1435265_at
1435280_at
1435315_s_at
1435331_at
1435454_a_at
1435477_s_at
1435529_at
1435551_at
1435560_at
1435582_at
1435584_at
1435595_at
1435621_at
1435665_at
1435719_at
1435792_at
1435906_x_at
1435933_at
1436058_at
1436160_at
1436171_at
1436172_at
1436199_at

1436482_a_at
1436530_at
1436590_at
1436625_at
1436659_at
1436671_at
1436722_a_at
1436763_a_at
1436778_at
1436779_at
1436838_x_at
1436871_at
1436873_at
1436902_x_at
1436996_x_at
1436999_at
1437024_at
1437072_at
1437129_at
1437218_at
1437234_x_at
1437440_at
1437514_at
1437570_at
1437811_x_at
1437899_at
1438037_at
1438052_at
1438075_at
1438148_at
1438220_at
1438295_at
1438566_at
1438651_a_at
1438704_at

1438707_at
1438768_at
1438800_at
1438862_at
1438868_at
1438896_at
1438931_s_at
1438980_x_at
1438989_s_at
1439016_x_at
1439030_at
1439081_at
1439100_s_at
1439426_x_at
1439494_at
1439622_at
1439774_at
1439793_at
1439806_at
1439814_at
1439825_at
1439902_at
1439912_at
1439947_at
1439948_at
1439956_at
1440007_at
1440037_at
1440150_at
1440169_x_at
1440196_at
1440225_at
1440226_at
1440298_at
1440311_at

1440342_at
1440461_at
1440635_at
1440719_at
1440721_at
1440852_at
1440865_at
1440866_at
1440986_at
1441094_at
1441189_at
1441307_at
1441376_at
1441444_at
1441445_at
1441887_x_at
1442018_at
1442025_a_at
1442026_at
1442074_at
1442082_at
1442089_at
1442233_at
1442257_at
1442393_at
1442425_at
1442461_at
1442804_at
1442977_at
1443043_at
1443116_at
1443128_at
1443235_at
1443338_at
1443673_x_at

1443698_at
1443771_x_at
1443894_at
1443962_at
1443983_at
1444195_at
1444226_at
1444376_at
1444456_at
1444546_at
1444559_at
1444599_at
1445104_at
1445518_at
1445687_at
1445882_at
1446001_at
1446269_at
1446609_at
1446684_at
1446693_at
1446921_at
1447181_s_at
1447213_at
1447329_at
1447517_at
1447527_at
1447584_s_at
1447621_s_at
1448025_at
1448061_at
1448160_at
1448162_at
1448181_at
1448291_at

1448301_s_at
1448325_at
1448397_at
1448507_at
1448529_at
1448534_at
1448561_at
1448575_at
1448576_at
1448591_at
1448617_at
1448620_at
1448632_at
1448700_at
1448710_at
1448731_at
1448747_at
1448748_at
1448749_at
1448756_at
1448797_at
1448877_at
1448883_at
1448898_at
1448929_at
1449009_at
1449025_at
1449049_at
1449124_at
1449127_at
1449135_at
1449153_at
1449164_at
1449175_at
1449176_a_at

1449254_at
1449305_at
1449360_at
1449366_at
1449387_at
1449399_a_at
1449451_at
1449453_at
1449454_at
1449455_at
1449461_at
1449556_at
1449559_at
1449560_at
1449591_at
1449824_at
1449858_at
1449874_at
1449919_at
1449945_at
1449963_at
1449976_a_at
1449981_a_at
1449984_at
1449986_at
1450027_at
1450033_a_at
1450034_at
1450065_at
1450135_at
1450165_at
1450170_x_at
1450188_s_at
1450199_a_at
1450234_at

1450241_a_at
1450291_s_at
1450297_at
1450322_s_at
1450379_at
1450403_at
1450454_at
1450484_a_at
1450505_a_at
1450508_at
1450517_at
1450536_s_at
1450616_at
1450618_a_at
1450639_at
1450645_at
1450678_at
1450696_at
1450774_at
1450783_at
1450788_at
1450792_at
1450808_at
1450826_a_at
1450843_a_at
1450871_a_at
1450919_at
1450967_at
1451156_s_at
1451161_a_at
1451174_at
1451318_a_at
1451335_at
1451362_at
1451382_at

1451426_at
1451564_at
1451567_a_at
1451634_at
1451655_at
1451767_at
1451774_at
1451777_at
1451798_at
1451859_at
1451860_a_at
1451905_a_at
1451941_a_at
1451956_a_at
1452016_at
1452087_at
1452117_a_at
1452126_at
1452203_at
1452261_at
1452279_at
1452348_s_at
1452349_x_at
1452382_at
1452487_x_at
1452527_a_at
1452719_at
1452948_at
1452957_at
1453196_a_at
1453247_at
1453287_at
1453332_at
1453410_at
1453503_at

1453523_at
1453591_at
1453628_s_at
1453898_at
1454018_at
1454169_a_at
1454242_at
1454268_a_at
1454646_at
1454699_at
1454867_at
1454878_at
1455058_at
1455065_x_at
1455203_at
1455238_at
1455269_a_at
1455332_x_at
1455418_at
1455419_at
1455500_at
1455573_at
1455581_x_at
1455660_at
1455860_at
1455899_x_at
1456001_at
1456014_s_at
1456047_at
1456103_at
1456147_at
1456307_s_at
1456331_at
1456341_a_at
1456377_x_at

1456440_s_at
1456494_a_at
1456733_x_at
1456772_at
1456815_at
1456890_at
1456901_at
1456944_at
1457042_at
1457117_at
1457228_x_at
1457644_s_at
1457666_s_at
1457753_at
1457967_at
1458053_at
1458299_s_at
1458354_x_at
1458426_at
1458467_at
1458680_at
1459003_at
1459219_at
1459760_at
1459823_at
1460020_at
1460036_at
1460185_at
1460188_at
1460218_at
1460227_at
1460259_s_at
1460283_at
1460336_at
1460437_at

1460603_at
1460604_at
1460668_at
