# Supplementary material for: Fibroblasts accelerate islet revascularization and improve long-term graft survival in a mouse model of subcutaneous islet transplantation
Source: PLoS One. 2017 Jul 3;12(7):e0180695. doi: 10.1371/journal.pone.0180695 (PMC5495486; doi:10.1371/journal.pone.0180695)
Supplement: S2 Table — (PDF) [file pone.0180695.s004.pdf]

Day 3

Prob SET_ID

Specific PSI-F genes	Specific PSI genes	Common genes
1415780_a_at	1416067_at	1415712_at
1415806_at	1416505_at	1415951_at
1415836_at	1416897_at	1415983_at
1415877_at	1417025_at	1416151_at
1415893_at	1417141_at	1416152_a_at
1415905_at	1417244_a_at	1416191_at
1415906_at	1417292_at	1416200_at
1415923_at	1417821_at	1416226_at
1415989_at	1417898_a_at	1416246_a_at
1415994_at	1418126_at	1416295_a_at
1416002_x_at	1418191_at	1416296_at
1416035_at	1418240_at	1416318_at
1416188_at	1418293_at	1416342_at
1416190_a_at	1418392_a_at	1416356_at
1416273_at	1418534_at	1416407_at
1416298_at	1418580_at	1416414_at
1416325_at	1418652_at	1416527_at
1416326_at	1418769_at	1416617_at
1416340_a_at	1418776_at	1416686_at
1416382_at	1418979_at	1416687_at
1416390_at	1419220_at	1416714_at
1416548_at	1419603_at	1416740_at
1416559_at	1419604_at	1416741_at
1416572_at	1419605_at	1416871_at
1416576_at	1420575_at	1416935_at
1416613_at	1420582_at	1416956_at
1416627_at	1420936_s_at	1416985_at
1416776_at	1422160_at	1416986_a_at
1416818_at	1422558_at	1417018_at

1416832_at	1422672_at	1417070_at
1416926_at	1422908_at	1417104_at
1416981_at	1424775_at	1417219_s_at
1416983_s_at	1424923_at	1417266_at
1417045_at	1425065_at	1417273_at
1417069_a_at	1425374_at	1417314_at
1417092_at	1425394_at	1417381_at
1417130_s_at	1426005_at	1417392_a_at
1417234_at	1426278_at	1417426_at
1417256_at	1426415_a_at	1417439_at
1417262_at	1426464_at	1417522_at
1417263_at	1428111_at	1417523_at
1417267_s_at	1428615_at	1417620_at
1417268_at	1429947_a_at	1417634_at
1417272_at	1430126_at	1417676_a_at
1417391_a_at	1430295_at	1417793_at
1417601_at	1431591_s_at	1417813_at
1417633_at	1431830_at	1417876_at
1417649_at	1431836_x_at	1417936_at
1417653_at	1433902_at	1417961_a_at
1417697_at	1433933_s_at	1417963_at
1417741_at	1434139_at	1417976_at
1417786_a_at	1434372_at	1418008_at
1417802_at	1434438_at	1418084_at
1417803_at	1434618_at	1418099_at
1417836_at	1435603_at	1418204_s_at
1418028_at	1435621_at	1418248_at
1418061_at	1435665_at	1418261_at
1418069_at	1435792_at	1418288_at
1418076_at	1435906_x_at	1418323_at
1418091_at	1436067_at	1418340_at
1418133_at	1436202_at	1418344_at
1418162_at	1436482_a_at	1418345_at

1418172_at
1418173_at
1418174_at
1418262_at
1418269_at
1418296_at
1418318_at
1418364_a_at
1418492_at
1418589_a_at
1418595_at
1418616_at
1418678_at
1418706_at
1418709_at
1418742_at
1418755_at
1418806_at
1418815_at
1418829_a_at
1418852_at
1418895_at
1418951_at
1418989_at
1418992_at
1419062_at
1419098_at
1419100_at
1419128_at
1419132_at
1419146_a_at
1419193_a_at
1419219_at
1436576_at
1436625_at
1437162_at
1438037_at
1438239_at
1439041_at
1439221_s_at
1439831_at
1440202_at
1442484_at
1442632_at
1442745_x_at
1443167_at
1443962_at
1444487_at
1444599_at
1446521_at
1446921_at
1448452_at
1448576_at
1449130_at
1449131_s_at
1449446_at
1449556_at
1449580_s_at
1450027_at
1450033_a_at
1450170_x_at
1450454_at
1450696_at
1450783_at
1451348_at
1451426_at
1418379_s_at
1418396_at
1418402_at
1418424_at
1418440_at
1418465_at
1418480_at
1418483_a_at
1418509_at
1418538_at
1418547_at
1418626_a_at
1418641_at
1418666_at
1418697_at
1418736_at
1418747_at
1418809_at
1418826_at
1418842_at
1418932_at
1418936_at
1418944_at
1418945_at
1418946_at
1419004_s_at
1419042_at
1419043_a_at
1419099_x_at
1419120_at
1419125_at
1419149_at
1419186_a_at

1419249_at
1419295_at
1419323_at
1419327_at
1419449_a_at
1419463_at
1419507_at
1419509_a_at
1419526_at
1419534_at
1419555_at
1419586_at
1419589_at
1419591_at
1419610_at
1419693_at
1419707_at
1419709_at
1419767_at
1419810_x_at
1419840_at
1419971_s_at
1420331_at
1420346_at
1420358_at
1420382_at
1420409_at
1420413_at
1420538_at
1420558_at
1420599_at
1420653_at
1420674_at

1451564_at
1451905_a_at
1451989_a_at
1452067_at
1452358_at
1452417_x_at
1452500_at
1452610_at
1452815_at
1453136_at
1453571_at
1454283_at
1455493_at
1455500_at
1455679_at
1455778_at
1456424_s_at
1456475_s_at
1456676_a_at
1457692_at
1458065_at
1458426_at
1458585_at

1419194_s_at
1419206_at
1419209_at
1419282_at
1419309_at
1419321_at
1419394_s_at
1419431_at
1419455_at
1419474_a_at
1419480_at
1419482_at
1419483_at
1419487_at
1419519_at
1419537_at
1419549_at
1419561_at
1419573_a_at
1419587_s_at
1419598_at
1419599_s_at
1419609_at
1419627_s_at
1419631_at
1419684_at
1419692_a_at
1419703_at
1419714_at
1419728_at
1419764_at
1419872_at
1419873_s_at

1420686_at
1420693_at
1420728_at
1420779_at
1420819_at
1420991_at
1420992_at
1421034_a_at
1421355_at
1421366_at
1421457_a_at
1421589_at
1421691_at
1421698_a_at
1421775_at
1421813_a_at
1421856_at
1421871_at
1422209_s_at
1422301_at
1422302_s_at
1422498_at
1422542_at
1422588_at
1422639_at
1422743_at
1422744_at
1422755_at
1422771_at
1422783_a_at
1422784_at
1422808_s_at
1422831_at

1419874_x_at
1420161_at
1420249_s_at
1420310_at
1420330_at
1420361_at
1420380_at
1420394_s_at
1420398_at
1420415_at
1420464_s_at
1420465_s_at
1420498_a_at
1420671_x_at
1420697_at
1420699_at
1420703_at
1420751_at
1420804_s_at
1420915_at
1420970_at
1421074_at
1421075_s_at
1421106_at
1421171_at
1421172_at
1421186_at
1421187_at
1421188_at
1421228_at
1421326_at
1421408_at
1421492_at

1422852_at
1422868_s_at
1423017_a_at
1423100_at
1423135_at
1423174_a_at
1423175_s_at
1423267_s_at
1423326_at
1423327_at
1423350_at
1423396_at
1423407_a_at
1423478_at
1423590_at
1423593_a_at
1423606_at
1423607_at
1423635_at
1423693_at
1423753_at
1423860_at
1423909_at
1423915_at
1423996_a_at
1424099_at
1424157_at
1424234_s_at
1424254_at
1424265_at
1424338_at
1424382_at
1424495_a_at

1421525_a_at
1421551_s_at
1421596_s_at
1421644_at
1421689_at
1421694_a_at
1421792_s_at
1421839_at
1421977_at
1422013_at
1422046_at
1422062_at
1422124_a_at
1422153_a_at
1422190_at
1422191_at
1422264_s_at
1422317_a_at
1422341_s_at
1422430_at
1422437_at
1422446_x_at
1422447_at
1422562_at
1422570_at
1422601_at
1422628_at
1422875_at
1422903_at
1422932_a_at
1422953_at
1422973_a_at
1422977_at

1424526_a_at
1424542_at
1424650_at
1424692_at
1424717_at
1424824_at
1424831_at
1424832_at
1424906_at
1425002_at
1425145_at
1425237_at
1425431_at
1425567_a_at
1425575_at
1425603_at
1425801_x_at
1425850_a_at
1425874_at
1425942_a_at
1425958_at
1425967_a_at
1426010_a_at
1426025_s_at
1426037_a_at
1426165_a_at
1426180_a_at
1426221_at
1426306_a_at
1426314_at
1426383_at
1426642_at
1426725_s_at

1422978_at
1423140_at
1423141_at
1423155_at
1423182_at
1423294_at
1423321_at
1423547_at
1423555_a_at
1423569_at
1423570_at
1423672_at
1423704_at
1423768_at
1423805_at
1423858_a_at
1424033_at
1424131_at
1424211_at
1424271_at
1424302_at
1424312_at
1424349_a_at
1424375_s_at
1424443_at
1424524_at
1424552_at
1424556_at
1424659_at
1424683_at
1424713_at
1424727_at
1424737_at

1426958_at
1427007_at
1427045_at
1427055_at
1427118_at
1427179_at
1427180_at
1427211_at
1427290_at
1427298_at
1427327_at
1427329_a_at
1427364_a_at
1427365_at
1427366_at
1427378_at
1427483_at
1427556_at
1427700_x_at
1427719_s_at
1427751_a_at
1427883_a_at
1428007_at
1428081_at
1428130_at
1428288_at
1428306_at
1428352_at
1428357_at
1428393_at
1428420_a_at
1428444_at
1428485_at

1424754_at
1424795_a_at
1424807_at
1424927_at
1424938_at
1424965_at
1425001_at
1425025_at
1425099_a_at
1425133_s_at
1425214_at
1425225_at
1425282_at
1425303_at
1425407_s_at
1425420_s_at
1425430_at
1425435_at
1425451_s_at
1425548_a_at
1425598_a_at
1425609_at
1425662_at
1425829_a_at
1425860_x_at
1425863_a_at
1425872_at
1425894_at
1425896_a_at
1425917_at
1425951_a_at
1426039_a_at
1426154_s_at

1428538_s_at
1428636_at
1428733_at
1428767_at
1428835_at
1428854_at
1428902_at
1428926_at
1428988_at
1429065_at
1429104_at
1429180_at
1429215_at
1429219_at
1429297_at
1429298_at
1429300_at
1429413_at
1429530_a_at
1429598_at
1429778_at
1429944_at
1429957_at
1430036_at
1430132_at
1430332_a_at
1430357_at
1430460_at
1430509_at
1430604_a_at
1430612_at
1430635_at
1430669_at

1426203_at
1426454_at
1426501_a_at
1426505_at
1426516_a_at
1426536_at
1426604_at
1426806_at
1426971_at
1427041_at
1427056_at
1427072_at
1427076_at
1427102_at
1427200_at
1427256_at
1427301_at
1427321_s_at
1427339_at
1427381_at
1427388_at
1427540_at
1427549_s_at
1427566_at
1427747_a_at
1427884_at
1427892_at
1427994_at
1428018_a_at
1428083_at
1428129_at
1428289_at
1428294_at

1430703_at
1430731_at
1431171_at
1431504_at
1431650_at
1431724_a_at
1431805_a_at
1431843_a_at
1432001_at
1432129_a_at
1432331_a_at
1432352_at
1432517_a_at
1432540_at
1432885_at
1433147_at
1433398_at
1433512_at
1433596_at
1433711_s_at
1433719_at
1433877_at
1434013_at
1434061_at
1434067_at
1434068_s_at
1434069_at
1434130_at
1434190_at
1434316_at
1434362_at
1434425_at
1434430_s_at

1428391_at
1428392_at
1428492_at
1428699_at
1428748_at
1428786_at
1428787_at
1428903_at
1429140_at
1429169_at
1429184_at
1429235_at
1429344_at
1429524_at
1429525_s_at
1429570_at
1429637_at
1429679_at
1429693_at
1429775_a_at
1429831_at
1429866_at
1429914_at
1429918_at
1429954_at
1430030_at
1430172_a_at
1430379_at
1430447_a_at
1430462_at
1430579_at
1430581_at
1430584_s_at

1434542_at
1434628_a_at
1434893_at
1434940_x_at
1435043_at
1435105_at
1435110_at
1435125_at
1435134_at
1435143_at
1435154_at
1435184_at
1435190_at
1435280_at
1435343_at
1435364_at
1435370_a_at
1435373_at
1435382_at
1435383_x_at
1435468_at
1435551_at
1435567_at
1435584_at
1435585_at
1435605_at
1435639_at
1435679_at
1435761_at
1435893_at
1435933_at
1435945_a_at
1436001_at

1430623_s_at
1430655_at
1430700_a_at
1431004_at
1431094_at
1431166_at
1431182_at
1431394_a_at
1431705_a_at
1433434_at
1433465_a_at
1433466_at
1433617_s_at
1433678_at
1433741_at
1433836_a_at
1433837_at
1433930_at
1433963_a_at
1434099_at
1434100_x_at
1434110_x_at
1434129_s_at
1434202_a_at
1434350_at
1434380_at
1434413_at
1434457_at
1434479_at
1434955_at
1434980_at
1435059_at
1435144_at

1436074_at
1436119_at
1436160_at
1436193_at
1436236_x_at
1436309_at
1436312_at
1436319_at
1436361_at
1436398_at
1436431_at
1436532_at
1436557_at
1436671_at
1436722_a_at
1436873_at
1436905_x_at
1436913_at
1436999_at
1437024_at
1437121_at
1437152_at
1437185_s_at
1437234_x_at
1437303_at
1437440_at
1437445_at
1437451_at
1437458_x_at
1437570_at
1437576_at
1437668_at
1437689_x_at
1435263_at
1435264_at
1435315_s_at
1435331_at
1435375_at
1435476_a_at
1435477_s_at
1435560_at
1435582_at
1435595_at
1435719_at
1435903_at
1436037_at
1436171_at
1436172_at
1436199_at
1436397_at
1436453_at
1436530_at
1436590_at
1436594_at
1436659_at
1436763_a_at
1436778_at
1436779_at
1436838_x_at
1436871_at
1436902_x_at
1436996_x_at
1437129_at
1437218_at
1437245_at
1437401_at

1437718_x_at
1437751_at
1437788_at
1437873_at
1437899_at
1437902_s_at
1437904_at
1438130_at
1438202_at
1438211_s_at
1438220_at
1438345_at
1438385_s_at
1438405_at
1438483_at
1438531_at
1438566_at
1438608_at
1438639_x_at
1438676_at
1438707_at
1438768_at
1438796_at
1438855_x_at
1438862_at
1438980_x_at
1438989_s_at
1439015_at
1439016_x_at
1439030_at
1439100_s_at
1439148_a_at
1439263_at

1437514_at
1437811_x_at
1438052_at
1438059_at
1438075_at
1438104_at
1438148_at
1438295_at
1438475_at
1438651_a_at
1438702_at
1438704_at
1438775_at
1438800_at
1438896_at
1438931_s_at
1439081_at
1439163_at
1439173_at
1439426_x_at
1439622_at
1439774_at
1439814_at
1439902_at
1439912_at
1439948_at
1439956_at
1440007_at
1440037_at
1440169_x_at
1440196_at
1440226_at
1440635_at

1439389_s_at
1439494_at
1439589_at
1439675_at
1439764_s_at
1439793_at
1439806_at
1439836_at
1439947_at
1440010_at
1440123_at
1440150_at
1440225_at
1440250_at
1440311_at
1440441_at
1440840_at
1440985_at
1441020_at
1441056_at
1441094_at
1441111_at
1441307_at
1441516_a_at
1441991_at
1442089_at
1442116_at
1442118_at
1442169_at
1442257_at
1442339_at
1442425_at
1442804_at
1440719_at
1440721_at
1440852_at
1441083_at
1441189_at
1441376_at
1441445_at
1442018_at
1442025_a_at
1442026_at
1442082_at
1442393_at
1442461_at
1443116_at
1443128_at
1443235_at
1443745_s_at
1443858_at
1443894_at
1443949_at
1444195_at
1444226_at
1444376_at
1444456_at
1444546_at
1445104_at
1445381_at
1445518_at
1445882_at
1446001_at
1446269_at
1446609_at
1446684_at

1442977_at
1443043_at
1443323_at
1443558_s_at
1443591_at
1443771_x_at
1443960_at
1443983_at
1444061_at
1444289_at
1444447_at
1444493_at
1444559_at
1445687_at
1446326_at
1447116_at
1447284_at
1447670_at
1447713_at
1447830_s_at
1447852_x_at
1447927_at
1448201_at
1448250_at
1448276_at
1448291_at
1448326_a_at
1448377_at
1448407_at
1448449_at
1448457_at
1448470_at
1448484_at
1446693_at
1447181_s_at
1447213_at
1447329_at
1447517_at
1447527_at
1447584_s_at
1447621_s_at
1448025_at
1448061_at
1448124_at
1448160_at
1448162_at
1448163_at
1448181_at
1448183_a_at
1448239_at
1448259_at
1448301_s_at
1448316_at
1448325_at
1448534_at
1448550_at
1448561_at
1448575_at
1448591_at
1448594_at
1448617_at
1448620_at
1448700_at
1448710_at
1448731_at
1448732_at

1448485_at
1448507_at
1448593_at
1448601_s_at
1448605_at
1448636_at
1448648_at
1448742_at
1448756_at
1448816_at
1448872_at
1448877_at
1448881_at
1448932_at
1449036_at
1449091_at
1449169_at
1449227_at
1449305_at
1449356_at
1449366_at
1449378_at
1449387_at
1449401_at
1449451_at
1449456_a_at
1449559_at
1449560_at
1449591_at
1449632_s_at
1449755_at
1449851_at
1449856_at
1448747_at
1448748_at
1448749_at
1448797_at
1448823_at
1448883_at
1448894_at
1448898_at
1448901_at
1448929_at
1448943_at
1448944_at
1448995_at
1449009_at
1449049_at
1449124_at
1449127_at
1449135_at
1449153_at
1449164_at
1449175_at
1449193_at
1449254_at
1449282_at
1449310_at
1449360_at
1449399_a_at
1449453_at
1449454_at
1449455_at
1449461_at
1449858_at
1449874_at

1449859_at
1449865_at
1449873_at
1449901_a_at
1449919_at
1449963_at
1449986_at
1449996_a_at
1449997_at
1450014_at
1450105_at
1450136_at
1450162_at
1450165_at
1450214_at
1450259_a_at
1450322_s_at
1450377_at
1450392_at
1450449_a_at
1450468_at
1450475_at
1450508_at
1450536_s_at
1450618_a_at
1450629_at
1450645_at
1450734_at
1450757_at
1450774_at
1450779_at
1450808_at
1450919_at

1449945_at
1449976_a_at
1449981_a_at
1449984_at
1450034_at
1450047_at
1450065_at
1450135_at
1450199_a_at
1450234_at
1450241_a_at
1450291_s_at
1450297_at
1450379_at
1450430_at
1450505_a_at
1450616_at
1450625_at
1450639_at
1450641_at
1450678_at
1450792_at
1450826_a_at
1450843_a_at
1450871_a_at
1450872_s_at
1450967_at
1451156_s_at
1451161_a_at
1451174_at
1451318_a_at
1451335_at
1451353_at

1450939_at
1450958_at
1451097_at
1451203_at
1451289_at
1451362_at
1451374_x_at
1451415_at
1451537_at
1451563_at
1451774_at
1451775_s_at
1451798_at
1451978_at
1452093_at
1452163_at
1452277_at
1452352_at
1452410_a_at
1452433_at
1452483_a_at
1452487_x_at
1452504_s_at
1452521_a_at
1452595_at
1452803_at
1452857_at
1452861_at
1452881_at
1452913_at
1452957_at
1453009_at
1453247_at

1451382_at
1451478_at
1451567_a_at
1451648_a_at
1451655_at
1451716_at
1451755_a_at
1451762_a_at
1451767_at
1451777_at
1451859_at
1451860_a_at
1451886_at
1451941_a_at
1451956_a_at
1452014_a_at
1452016_at
1452087_at
1452117_a_at
1452126_at
1452191_at
1452203_at
1452261_at
1452279_at
1452345_at
1452348_s_at
1452349_x_at
1452382_at
1452436_at
1452527_a_at
1452707_at
1452719_at
1452948_at

1453344_at
1453355_at
1453410_at
1453523_at
1453775_at
1453898_at
1454242_at
1454713_s_at
1454734_at
1454764_s_at
1454806_at
1454830_at
1455007_s_at
1455030_at
1455094_s_at
1455136_at
1455160_at
1455161_at
1455220_at
1455226_at
1455267_at
1455271_at
1455396_at
1455419_at
1455426_at
1455455_at
1455573_at
1455627_at
1455737_at
1455899_x_at
1456046_at
1456047_at
1456064_at

1452968_at
1453196_a_at
1453287_at
1453332_at
1453455_at
1453503_at
1453591_at
1453628_s_at
1454018_at
1454169_a_at
1454240_at
1454268_a_at
1454613_at
1454699_at
1454768_at
1454849_x_at
1454867_at
1454878_at
1455050_at
1455058_at
1455065_x_at
1455096_at
1455269_a_at
1455332_x_at
1455377_at
1455418_at
1455439_a_at
1455473_at
1455660_at
1455860_at
1456014_s_at
1456307_s_at
1456331_at

1456147_at
1456212_x_at
1456389_at
1456395_at
1456404_at
1456440_s_at
1456706_at
1456739_x_at
1456815_at
1456893_at
1456901_at
1456929_at
1456944_at
1456968_at
1457231_at
1457266_at
1457586_at
1457644_s_at
1457721_at
1457881_at
1457967_at
1458354_x_at
1458660_at
1458680_at
1458781_at
1458919_at
1459170_at
1459238_at
1459557_at
1459622_at
1460014_at
1460185_at
1460202_at

1456341_a_at
1456344_at
1456377_x_at
1456442_at
1456733_x_at
1456772_at
1457042_at
1457117_at
1457228_x_at
1457243_at
1457666_s_at
1457753_at
1457779_at
1458299_s_at
1458382_a_at
1458467_at
1459202_at
1459219_at
1459760_at
1459823_at
1460020_at
1460036_at
1460188_at
1460197_a_at
1460208_at
1460218_at
1460227_at
1460283_at
1460336_at
1460377_a_at
1460419_a_at
1460437_at
1460603_at

1460220_a_at
1460253_at
1460259_s_at
1460273_a_at
1460281_at
1460302_at
1460347_at
1460600_at
1460604_at
AFFX-b-ActinMur/M12481_M_at
