# Supplementary material for: Fibroblasts accelerate islet revascularization and improve long-term graft survival in a mouse model of subcutaneous islet transplantation
Source: PLoS One. 2017 Jul 3;12(7):e0180695. doi: 10.1371/journal.pone.0180695 (PMC5495486; doi:10.1371/journal.pone.0180695)
Supplement: S3 Table — (PDF) [file pone.0180695.s005.pdf]

Day 7

Prob SET_ID

Specific PSI-F genes	Specific PSI genes	Common genes
1415712_at	1415806_at	1415780_a_at
1415906_at	1415832_at	1415923_at
1415996_at	1415861_at	1415951_at
1415997_at	1415862_at	1415983_at
1416002_x_at	1415927_at	1416067_at
1416191_at	1415972_at	1416111_at
1416332_at	1416006_at	1416125_at
1416340_a_at	1416151_at	1416129_at
1416617_at	1416652_at	1416200_at
1416832_at	1416776_at	1416246_a_at
1416875_at	1416803_at	1416295_a_at
1416897_at	1416805_at	1416296_at
1416926_at	1417092_at	1416342_at
1416982_at	1417234_at	1416356_at
1416983_s_at	1417267_s_at	1416390_at
1417172_at	1417359_at	1416405_at
1417244_a_at	1417599_at	1416407_at
1417381_at	1417600_at	1416414_at
1417392_a_at	1417601_at	1416454_s_at
1417639_at	1417821_at	1416572_at
1417898_a_at	1417851_at	1416589_at
1417902_at	1417943_at	1416687_at
1417961_a_at	1418008_at	1416714_at
1417963_at	1418063_at	1416740_at
1417976_at	1418173_at	1416741_at
1418100_at	1418174_at	1416818_at
1418261_at	1418282_x_at	1416871_at
1418269_at	1418283_at	1416882_at
1418293_at	1418424_at	1416981_at

1418344_at
1418396_at
1418432_at
1418480_at
1418487_at
1418488_s_at
1418493_a_at
1418718_at
1418747_at
1418769_at
1418825_at
1418842_at
1418887_a_at
1418930_at
1419015_at
1419060_at
1419132_at
1419220_at
1419309_at
1419369_at
1419404_s_at
1419449_a_at
1419480_at
1419485_at
1419486_at
1419492_s_at
1419505_a_at
1419537_at
1419569_a_at
1419603_at
1419604_at
1419631_at
1419697_at
1418450_at
1418454_at
1418483_a_at
1418492_at
1418571_at
1418616_at
1418742_at
1418829_a_at
1418945_at
1418979_at
1418989_at
1419209_at
1419249_at
1419323_at
1419463_at
1419487_at
1419507_at
1419591_at
1419662_at
1419707_at
1419709_at
1419767_at
1419840_at
1420358_at
1420465_s_at
1420538_at
1420599_at
1420674_at
1420855_at
1420940_x_at
1420970_at
1420994_at
1421002_at
1416985_at
1417018_at
1417025_at
1417063_at
1417070_at
1417104_at
1417141_at
1417219_s_at
1417256_at
1417262_at
1417272_at
1417273_at
1417281_a_at
1417282_at
1417292_at
1417314_at
1417439_at
1417522_at
1417523_at
1417620_at
1417676_a_at
1417793_at
1417802_at
1417803_at
1417813_at
1417836_at
1417876_at
1418061_at
1418084_at
1418099_at
1418126_at
1418136_at
1418191_at

1419714_at
1419762_at
1420398_at
1420549_at
1420583_a_at
1420671_x_at
1420703_at
1421058_at
1421106_at
1421182_at
1421290_at
1421366_at
1421525_a_at
1421551_s_at
1421792_s_at
1421977_at
1422062_at
1422143_at
1422340_a_at
1422412_x_at
1422527_at
1422557_s_at
1422560_at
1422633_at
1422932_a_at
1423141_at
1423182_at
1423375_at
1423858_a_at
1423862_at
1424034_at
1424296_at
1424349_a_at
1421045_at
1421385_a_at
1421424_a_at
1421492_at
1421589_at
1422209_s_at
1422430_at
1422571_at
1422580_at
1422831_at
1422908_at
1422977_at
1423037_at
1423100_at
1423136_at
1423155_at
1423261_at
1423321_at
1423365_at
1423439_at
1423554_at
1423584_at
1423608_at
1423635_at
1423672_at
1423860_at
1423909_at
1424010_at
1424032_at
1424083_at
1424157_at
1424374_at
1424659_at
1418204_s_at
1418240_at
1418288_at
1418323_at
1418340_at
1418345_at
1418379_s_at
1418392_a_at
1418402_at
1418440_at
1418465_at
1418534_at
1418538_at
1418547_at
1418572_x_at
1418580_at
1418599_at
1418626_a_at
1418641_at
1418652_at
1418697_at
1418736_at
1418776_at
1418809_at
1418815_at
1418826_at
1418932_at
1418936_at
1419004_s_at
1419042_at
1419043_a_at
1419120_at
1419125_at

1424409_at
1424724_a_at
1424857_a_at
1425065_at
1425156_at
1425225_at
1425374_at
1425394_at
1425407_s_at
1425425_a_at
1425477_x_at
1425480_at
1425801_x_at
1426025_s_at
1426040_a_at
1426348_at
1426383_at
1426464_at
1426521_at
1426970_a_at
1427007_at
1427042_at
1427135_at
1427271_at
1427381_at
1427397_at
1427511_at
1427660_x_at
1427866_x_at
1428081_at
1428111_at
1428288_at
1428572_at
1424692_at
1424824_at
1425227_a_at
1425431_at
1425567_a_at
1425603_at
1425681_a_at
1426005_at
1426039_a_at
1426154_s_at
1426208_x_at
1426225_at
1426536_at
1426758_s_at
1426852_x_at
1426864_a_at
1426871_at
1427056_at
1427115_at
1427118_at
1427179_at
1427211_at
1427256_at
1427290_at
1427344_s_at
1427365_at
1427366_at
1427378_at
1427751_a_at
1428007_at
1428259_at
1428615_at
1428721_at
1419128_at
1419146_a_at
1419149_at
1419186_a_at
1419194_s_at
1419282_at
1419295_at
1419321_at
1419431_at
1419474_a_at
1419482_at
1419483_at
1419491_at
1419519_at
1419561_at
1419573_a_at
1419589_at
1419598_at
1419599_s_at
1419609_at
1419627_s_at
1419684_at
1419703_at
1419728_at
1419816_s_at
1419872_at
1419874_x_at
1420097_at
1420161_at
1420330_at
1420361_at
1420380_at
1420394_s_at

1428699_at
1428742_at
1428767_at
1428939_s_at
1429050_at
1429216_at
1429247_at
1429251_at
1429530_a_at
1429759_at
1429947_a_at
1430114_at
1430357_at
1430462_at
1430575_a_at
1430576_at
1431176_at
1431591_s_at
1431805_a_at
1431843_a_at
1433617_s_at
1433863_at
1433935_at
1434069_at
1434359_at
1434438_at
1434542_at
1434927_at
1435169_at
1435205_at
1435264_at
1435265_at
1435316_at
1428748_at
1428922_at
1429065_at
1429219_at
1429300_at
1429344_at
1429802_at
1429866_at
1429957_at
1429960_at
1430132_at
1430388_a_at
1430509_at
1430623_s_at
1430655_at
1430669_at
1430703_at
1430731_at
1431079_at
1431094_at
1431394_a_at
1431504_at
1431650_at
1431836_x_at
1432540_at
1432885_at
1433512_at
1433600_at
1434186_at
1434202_a_at
1434325_x_at
1434425_at
1434740_at
1420415_at
1420464_s_at
1420498_a_at
1420504_at
1420686_at
1420697_at
1420699_at
1420728_at
1420731_a_at
1420751_at
1420804_s_at
1420915_at
1421074_at
1421075_s_at
1421171_at
1421172_at
1421187_at
1421188_at
1421228_at
1421326_at
1421408_at
1421596_s_at
1421689_at
1421691_at
1421694_a_at
1421856_at
1422013_at
1422124_a_at
1422301_at
1422341_s_at
1422437_at
1422446_x_at
1422447_at

1435331_at
1435364_at
1435476_a_at
1435562_at
1435665_at
1435792_at
1435945_a_at
1436058_at
1436199_at
1436202_at
1436309_at
1436326_at
1436431_at
1436722_a_at
1436853_a_at
1436905_x_at
1437071_at
1437100_x_at
1437665_at
1437676_at
1437689_x_at
1437726_x_at
1437751_at
1438027_at
1438037_at
1438052_at
1438220_at
1438325_at
1438345_at
1438473_at
1438475_at
1438663_at
1438775_at

1434848_at
1435021_at
1435059_at
1435120_at
1435143_at
1435162_at
1435315_s_at
1435373_at
1435382_at
1435409_at
1435468_at
1435584_at
1435585_at
1435872_at
1435940_at
1435952_at
1436025_at
1436160_at
1436188_a_at
1436713_s_at
1436873_at
1436898_at
1437303_at
1437440_at
1437873_at
1437889_x_at
1438202_at
1438211_s_at
1438238_at
1438707_at
1438768_at
1438800_at
1438931_s_at

1422542_at
1422558_at
1422570_at
1422588_at
1422601_at
1422606_at
1422628_at
1422875_at
1422903_at
1422953_at
1422973_a_at
1422978_at
1423110_at
1423140_at
1423169_at
1423174_a_at
1423175_s_at
1423233_at
1423294_at
1423396_at
1423407_a_at
1423516_a_at
1423547_at
1423555_a_at
1423569_at
1423606_at
1423607_at
1423669_at
1423704_at
1423768_at
1423915_at
1424033_at
1424084_at

1438855_x_at
1438868_at
1438900_at
1439080_at
1439081_at
1439221_s_at
1439316_at
1439638_at
1439819_at
1439831_at
1440007_at
1440085_at
1440092_at
1440225_at
1440250_at
1440903_at
1440990_at
1441231_at
1441233_at
1441346_at
1441410_at
1441956_s_at
1442044_at
1442535_at
1442804_at
1443109_at
1443167_at
1443621_at
1443698_at
1443960_at
1444180_at
1444447_at
1444599_at
1438989_s_at
1439016_x_at
1439100_s_at
1439163_at
1439380_x_at
1439389_s_at
1439556_at
1439665_at
1439793_at
1440226_at
1440355_at
1440852_at
1440866_at
1441108_at
1441307_at
1441779_at
1441793_at
1441799_at
1442089_at
1442461_at
1443128_at
1443323_at
1443558_s_at
1443745_s_at
1444061_at
1444494_at
1445391_at
1446048_at
1446190_at
1447669_s_at
1448424_at
1448457_at
1448475_at
1424099_at
1424131_at
1424211_at
1424234_s_at
1424271_at
1424302_at
1424312_at
1424375_s_at
1424382_at
1424394_at
1424443_at
1424524_at
1424552_at
1424556_at
1424650_at
1424683_at
1424713_at
1424727_at
1424737_at
1424754_at
1424775_at
1424795_a_at
1424807_at
1424923_at
1424927_at
1424965_at
1425001_at
1425025_at
1425099_a_at
1425133_s_at
1425214_at
1425237_at
1425282_at

1444787_at
1444790_at
1445225_at
1446167_at
1446504_at
1446684_at
1446802_at
1446921_at
1447116_at
1447211_at
1447551_x_at
1447706_at
1448021_at
1448169_at
1448239_at
1448250_at
1448380_at
1448433_a_at
1448436_a_at
1448452_at
1448470_at
1448534_at
1448561_at
1448601_s_at
1448632_at
1448883_at
1448952_at
1448995_at
1449025_at
1449065_at
1449193_at
1449195_s_at
1449227_at
1448648_at
1449154_at
1449378_at
1449387_at
1449396_at
1449451_at
1449531_at
1449556_at
1449859_at
1449986_at
1450085_at
1450170_x_at
1450379_at
1450618_a_at
1450663_at
1450734_at
1450774_at
1451047_at
1451342_at
1451344_at
1451415_at
1451446_at
1451475_at
1451513_x_at
1451774_at
1452114_s_at
1452183_a_at
1452261_at
1452595_at
1452670_at
1452719_at
1452957_at
1454240_at
1425303_at
1425420_s_at
1425430_at
1425519_a_at
1425548_a_at
1425575_at
1425598_a_at
1425662_at
1425860_x_at
1425872_at
1425894_at
1425896_a_at
1425905_at
1425917_at
1425951_a_at
1426203_at
1426306_a_at
1426413_at
1426454_at
1426505_at
1426516_a_at
1426604_at
1426642_at
1426725_s_at
1426806_at
1426865_a_at
1426947_x_at
1426971_at
1427041_at
1427076_at
1427102_at
1427168_a_at
1427200_at

1449446_at
1449461_at
1449473_s_at
1449500_at
1449580_s_at
1449733_s_at
1449851_at
1449856_at
1450027_at
1450135_at
1450165_at
1450234_at
1450353_at
1450417_a_at
1450511_at
1450648_s_at
1451077_at
1451335_at
1451426_at
1451439_at
1451601_a_at
1451655_at
1451675_a_at
1451721_a_at
1451723_at
1451886_at
1451905_a_at
1452016_at
1452026_a_at
1452067_at
1452117_a_at
1452160_at
1452279_at

1454242_at
1454613_at
1454878_at
1454965_at
1455050_at
1455058_at
1455090_at
1455096_at
1455422_x_at
1455700_at
1455860_at
1456001_at
1456047_at
1456147_at
1456442_at
1456475_s_at
1456676_a_at
1456705_at
1456736_x_at
1456815_at
1457145_at
1457227_at
1457429_s_at
1458407_s_at
1458551_at
1458680_at
1459557_at
1460049_s_at
1460121_at
1460185_at
1460359_at
1460419_a_at

1427298_at
1427301_at
1427327_at
1427339_at
1427388_at
1427391_a_at
1427539_a_at
1427540_at
1427549_s_at
1427566_at
1427719_s_at
1427883_a_at
1427884_at
1427892_at
1427919_at
1427994_at
1428018_a_at
1428057_a_at
1428083_at
1428273_at
1428294_at
1428306_at
1428352_at
1428391_at
1428492_at
1428786_at
1428787_at
1428926_at
1429006_s_at
1429104_at
1429140_at
1429184_at
1429215_at

1452298_a_at
1452345_at
1452348_s_at
1452415_at
1452440_at
1452500_at
1452606_at
1453012_at
1453119_at
1453214_at
1453251_at
1453571_at
1453628_s_at
1454018_at
1454646_at
1454849_x_at
1454863_at
1454867_at
1455007_s_at
1455094_s_at
1455136_at
1455165_at
1455171_at
1455351_at
1455500_at
1455785_at
1456014_s_at
1456103_at
1456156_at
1456211_at
1456347_at
1456395_at
1456659_at

1429235_at
1429297_at
1429298_at
1429379_at
1429413_at
1429524_at
1429525_s_at
1429570_at
1429637_at
1429679_at
1429775_a_at
1429831_at
1429914_at
1429918_at
1429944_at
1429954_at
1430126_at
1430295_at
1430447_a_at
1430460_at
1430579_at
1430581_at
1430584_s_at
1430585_at
1430635_at
1430700_a_at
1431004_at
1431166_at
1431171_at
1431856_a_at
1432331_a_at
1432352_at
1433434_at

1456706_at
1456875_at
1456907_at
1456968_at
1457198_at
1457302_at
1457321_at
1457666_s_at
1457742_at
1457823_at
1458053_at
1458065_at
1458305_at
1458586_at
1458660_at
1458919_at
1459170_at
1459202_at
1459238_at
1459391_at
1459434_at
1459601_at
1459609_at
1459707_at
1460025_at
1460036_at
1460253_at
1460318_at
1460441_at
AFFX-b-ActinMur/M12481_M_at
AFFX-DapX-5_at
AFFX-MURINE_b1_at
AFFX-r2-Bs-thr-5_s_at
1433596_at
1433678_at
1433711_s_at
1433716_x_at
1433741_at
1433837_at
1433877_at
1433930_at
1434099_at
1434100_x_at
1434129_s_at
1434130_at
1434139_at
1434195_at
1434316_at
1434350_at
1434372_at
1434380_at
1434411_at
1434413_at
1434457_at
1434479_at
1434628_a_at
1434817_s_at
1434955_at
1435125_at
1435144_at
1435263_at
1435271_at
1435375_at
1435477_s_at
1435560_at
1435582_at

AFFX-ThrX-5_at	1435595_at
	1435719_at
	1435906_x_at
	1436037_at
	1436171_at
	1436172_at
	1436236_x_at
	1436325_at
	1436453_at
	1436530_at
	1436576_at
	1436590_at
	1436659_at
	1436671_at
	1436763_a_at
	1436778_at
	1436779_at
	1436838_x_at
	1436871_at
	1436902_x_at
	1436996_x_at
	1437024_at
	1437129_at
	1437152_at
	1437162_at
	1437185_s_at
	1437218_at
	1437401_at
	1437570_at
	1437668_at
	1437811_x_at
	1437899_at
	1438059_at

1438075_at
1438295_at
1438404_at
1438531_at
1438651_a_at
1438704_at
1438796_at
1438814_at
1438862_at
1438896_at
1439426_x_at
1439557_s_at
1439622_at
1439643_at
1439774_at
1439790_at
1439806_at
1439814_at
1439827_at
1439912_at
1439947_at
1439948_at
1439956_at
1440010_at
1440037_at
1440123_at
1440169_x_at
1440311_at
1440347_at
1440435_at
1440441_at
1440635_at
1440719_at

1440720_s_at
1440721_at
1441056_at
1441083_at
1441094_at
1441189_at
1441315_s_at
1441376_at
1441445_at
1442018_at
1442025_a_at
1442026_at
1442082_at
1442116_at
1442140_at
1442169_at
1442393_at
1442425_at
1442977_at
1443043_at
1443116_at
1443235_at
1443673_x_at
1443858_at
1443894_at
1443962_at
1443983_at
1444195_at
1444226_at
1444376_at
1444456_at
1444546_at
1444559_at

1444740_at
1445365_at
1445381_at
1445457_at
1445866_at
1445882_at
1446001_at
1446269_at
1446326_at
1446521_at
1446693_at
1447213_at
1447517_at
1447527_at
1447584_s_at
1447621_s_at
1447852_x_at
1448025_at
1448061_at
1448124_at
1448160_at
1448162_at
1448163_at
1448181_at
1448201_at
1448259_at
1448316_at
1448323_a_at
1448325_at
1448383_at
1448575_at
1448590_at
1448591_at

1448593_at
1448594_at
1448617_at
1448620_at
1448700_at
1448710_at
1448729_a_at
1448731_at
1448747_at
1448748_at
1448749_at
1448797_at
1448823_at
1448877_at
1448894_at
1448901_at
1448943_at
1448944_at
1449009_at
1449049_at
1449127_at
1449130_at
1449131_s_at
1449135_at
1449153_at
1449164_at
1449175_at
1449244_at
1449254_at
1449360_at
1449399_a_at
1449401_at
1449455_at

1449559_at
1449560_at
1449632_s_at
1449755_at
1449858_at
1449874_at
1449901_a_at
1449919_at
1449945_at
1449963_at
1449984_at
1450014_at
1450033_a_at
1450034_at
1450047_at
1450065_at
1450199_a_at
1450241_a_at
1450291_s_at
1450429_at
1450505_a_at
1450536_s_at
1450625_at
1450641_at
1450645_at
1450678_at
1450696_at
1450757_at
1450783_at
1450792_at
1450826_a_at
1450843_a_at
1450857_a_at

1450871_a_at
1450872_s_at
1450905_at
1450967_at
1451069_at
1451156_s_at
1451161_a_at
1451174_at
1451289_at
1451318_a_at
1451319_at
1451353_at
1451382_at
1451478_at
1451564_at
1451567_a_at
1451762_a_at
1451767_at
1451777_at
1451859_at
1451860_a_at
1451941_a_at
1451978_at
1451989_a_at
1452014_a_at
1452087_at
1452126_at
1452163_at
1452203_at
1452250_a_at
1452382_at
1452417_x_at
1452431_s_at

1452436_at
1452527_a_at
1452707_at
1452948_at
1452968_at
1453009_at
1453136_at
1453191_at
1453196_a_at
1453287_at
1453332_at
1453410_at
1453455_at
1453470_a_at
1453503_at
1453523_at
1453588_at
1453590_at
1453591_at
1453775_at
1454169_a_at
1454268_a_at
1454699_at
1454768_at
1454830_at
1455065_x_at
1455269_a_at
1455332_x_at
1455399_at
1455418_at
1455426_at
1455439_a_at
1455455_at

1455627_at
1455660_at
1455773_at
1456046_at
1456307_s_at
1456341_a_at
1456344_at
1456377_x_at
1456440_s_at
1456514_at
1456733_x_at
1456739_x_at
1456772_at
1456901_at
1457024_x_at
1457042_at
1457117_at
1457228_x_at
1457586_at
1457753_at
1457777_at
1457779_at
1457967_at
1458299_s_at
1458354_x_at
1458426_at
1458467_at
1458603_at
1459622_at
1459749_s_at
1459760_at
1459823_at
1460020_at

1460188_at
1460208_at
1460218_at
1460227_at
1460283_at
1460336_at
1460365_a_at
1460437_at
1460463_at
1460603_at
AFFX-r2-Bs-dap-5_at
