# Supplementary material for: Fibroblasts accelerate islet revascularization and improve long-term graft survival in a mouse model of subcutaneous islet transplantation
Source: PLoS One. 2017 Jul 3;12(7):e0180695. doi: 10.1371/journal.pone.0180695 (PMC5495486; doi:10.1371/journal.pone.0180695)
Supplement: S4 Table — (PDF) [file pone.0180695.s006.pdf]

Day 10

Prob SET_ID

Specific PSI-F genes	Specific PSI genes	Common genes
1415927_at	1415832_at	1415983_at
1416067_at	1415972_at	1416111_at
1416077_at	1416125_at	1416151_at
1416356_at	1416191_at	1416152_a_at
1417419_at	1416250_at	1416200_at
1417961_a_at	1416342_at	1416246_a_at
1417985_at	1416527_at	1416295_a_at
1418008_at	1416871_at	1416296_at
1418174_at	1416873_a_at	1416325_at
1418580_at	1416985_at	1416572_at
1418616_at	1417141_at	1416589_at
1418979_at	1417256_at	1416714_at
1419075_s_at	1417262_at	1416740_at
1419487_at	1417272_at	1416741_at
1420357_s_at	1417292_at	1417018_at
1420452_at	1417601_at	1417025_at
1420465_s_at	1417602_at	1417219_s_at
1420599_at	1417697_at	1417273_at
1420841_at	1418240_at	1417314_at
1420970_at	1418379_s_at	1417359_at
1420992_at	1418392_a_at	1417381_at
1421001_a_at	1418424_at	1417439_at
1421253_at	1418440_at	1417522_at
1421290_at	1418538_at	1417620_at
1421424_a_at	1418638_at	1417676_a_at
1421494_at	1418930_at	1417793_at
1422153_a_at	1418945_at	1417813_at
1422209_s_at	1419146_a_at	1417876_at
1422301_at	1419149_at	1417898_a_at

1422527_at
1422892_s_at
1423142_a_at
1423172_at
1423439_at
1424680_at
1424775_at
1424857_a_at
1425431_at
1426460_a_at
1426726_at
1426758_s_at
1426947_x_at
1427102_at
1427364_a_at
1427540_at
1427549_s_at
1427556_at
1428111_at
1428294_at
1428444_at
1428699_at
1428719_at
1429140_at
1429169_at
1429478_at
1429736_at
1430000_at
1430126_at
1430575_a_at
1430669_at
1430703_at
1431292_a_at

1419194_s_at
1419209_at
1419327_at
1419591_at
1419605_at
1419697_at
1419700_a_at
1419709_at
1419714_at
1419734_at
1419762_at
1420097_at
1420330_at
1420380_at
1420421_s_at
1420549_at
1420657_at
1420772_a_at
1420779_at
1420955_at
1421171_at
1421172_at
1421228_at
1421262_at
1421575_at
1421653_a_at
1422134_at
1422831_at
1423100_at
1423607_at
1424524_at
1424807_at
1424923_at

1418099_at
1418126_at
1418191_at
1418204_s_at
1418288_at
1418323_at
1418340_at
1418345_at
1418465_at
1418534_at
1418547_at
1418641_at
1418652_at
1418697_at
1418769_at
1418776_at
1418809_at
1418932_at
1418936_at
1419004_s_at
1419042_at
1419043_a_at
1419120_at
1419128_at
1419186_a_at
1419220_at
1419282_at
1419321_at
1419431_at
1419482_at
1419483_at
1419598_at
1419599_s_at

1431650_at
1432001_at
1433593_at
1433902_at
1433919_at
1434359_at
1434618_at
1434817_s_at
1434976_x_at
1435271_at
1435331_at
1435562_at
1436431_at
1436438_s_at
1436713_s_at
1437024_at
1437162_at
1437245_at
1437284_at
1437629_at
1437676_at
1438211_s_at
1438483_at
1438768_at
1439033_at
1439041_at
1439440_x_at
1439948_at
1440085_at
1440225_at
1440635_at
1440852_at
1441108_at
1425281_a_at
1425282_at
1425655_at
1425863_a_at
1426383_at
1426454_at
1426642_at
1426851_a_at
1426852_x_at
1427168_a_at
1427298_at
1427397_at
1427511_at
1428306_at
1428352_at
1428492_at
1428786_at
1429300_at
1429334_at
1429525_s_at
1429570_at
1430152_at
1430392_at
1430485_at
1430576_at
1433836_a_at
1433837_at
1434203_at
1434362_at
1434380_at
1434413_at
1434542_at
1435459_at
1419609_at
1419621_at
1419627_s_at
1419684_at
1419703_at
1419874_x_at
1420161_at
1420361_at
1420394_s_at
1420464_s_at
1420697_at
1420699_at
1420855_at
1420915_at
1421074_at
1421187_at
1421188_at
1421214_at
1421326_at
1421596_s_at
1422013_at
1422124_a_at
1422264_s_at
1422341_s_at
1422430_at
1422437_at
1422446_x_at
1422447_at
1422570_at
1422601_at
1422606_at
1422628_at
1422903_at

1441307_at
1441444_at
1442089_at
1442411_at
1442542_at
1443167_at
1443621_at
1443960_at
1446155_at
1446156_at
1446521_at
1446921_at
1447116_at
1447329_at
1447852_x_at
1448325_at
1448575_at
1448700_at
1449130_at
1449131_s_at
1449356_at
1450449_a_at
1450511_at
1450648_s_at
1450788_at
1451319_at
1451323_at
1451564_at
1451774_at
1452183_a_at
1452358_at
1452474_a_at
1452857_at
1435621_at
1435906_x_at
1436066_at
1436236_x_at
1436361_at
1436722_a_at
1437065_at
1437303_at
1437665_at
1438052_at
1438238_at
1439163_at
1439221_s_at
1439819_at
1440292_at
1440720_s_at
1441083_at
1441544_at
1441545_at
1441549_at
1441799_at
1442336_at
1442393_at
1443520_at
1444494_at
1446129_at
1446244_at
1446730_at
1446834_at
1447411_at
1447517_at
1447584_s_at
1447830_s_at
1422973_a_at
1422978_at
1423233_at
1423294_at
1423396_at
1423547_at
1423555_a_at
1423606_at
1423669_at
1423672_at
1423768_at
1424033_at
1424131_at
1424211_at
1424302_at
1424305_at
1424312_at
1424375_s_at
1424443_at
1424552_at
1424556_at
1424683_at
1424713_at
1424727_at
1424737_at
1424754_at
1424795_a_at
1424927_at
1425001_at
1425025_at
1425099_a_at
1425214_at
1425272_at

1452905_at
1452948_at
1453012_at
1453136_at
1453836_a_at
1454018_at
1455065_x_at
1455094_s_at
1455165_at
1455267_at
1455342_at
1455377_at
1455473_at
1455493_at
1455500_at
1456475_s_at
1456676_a_at
1456706_at
1456736_x_at
1456875_at
1457587_at
1457742_at
1458125_at
1459219_at
1459948_at

1448201_at
1448316_at
1448594_at
1448617_at
1448620_at
1448731_at
1448748_at
1448823_at
1448949_at
1449254_at
1449461_at
1449556_at
1449851_at
1449858_at
1449939_s_at
1449984_at
1450033_a_at
1450170_x_at
1450172_at
1450297_at
1450379_at
1450757_at
1450871_a_at
1451161_a_at
1451318_a_at
1451415_at
1451859_at
1451956_a_at
1451989_a_at
1452067_at
1452163_at
1452527_a_at
1453435_a_at

1425303_at
1425394_at
1425420_s_at
1425477_x_at
1425519_a_at
1425548_a_at
1425662_at
1425681_a_at
1425738_at
1425860_x_at
1425894_at
1425896_a_at
1425905_at
1425917_at
1425951_a_at
1426039_a_at
1426074_at
1426505_at
1426516_a_at
1426536_at
1426604_at
1426727_s_at
1426806_at
1426971_at
1427041_at
1427045_at
1427076_at
1427200_at
1427301_at
1427321_s_at
1427327_at
1427339_at
1427351_s_at

1453455_at
1454830_at
1454881_s_at
1455007_s_at
1455773_at
1455889_at
1456331_at
1456377_x_at
1456442_at
1456514_at
1456741_s_at
1456907_at
1456968_at
1457447_at
1457579_at
1457823_at
1458065_at
1458426_at
1458660_at
1459557_at
1460121_at
1460227_at
1460463_at
AFFX-b-ActinMur/M12481_M_at

1427388_at
1427455_x_at
1427566_at
1427660_x_at
1427883_a_at
1427884_at
1427892_at
1427994_at
1428083_at
1428288_at
1428400_at
1428615_at
1428720_s_at
1428748_at
1428787_at
1428926_at
1429006_s_at
1429184_at
1429215_at
1429235_at
1429297_at
1429344_at
1429524_at
1429637_at
1429679_at
1429831_at
1429866_at
1429914_at
1429918_at
1429954_at
1430295_at
1430460_at
1430462_at

1430579_at
1430581_at
1430584_s_at
1430585_at
1430655_at
1431166_at
1433147_at
1433434_at
1433678_at
1433711_s_at
1434099_at
1434100_x_at
1434129_s_at
1434202_a_at
1434350_at
1434372_at
1434457_at
1434479_at
1434927_at
1435125_at
1435144_at
1435263_at
1435265_at
1435280_at
1435315_s_at
1435477_s_at
1435560_at
1435595_at
1435665_at
1435719_at
1435792_at
1436039_at
1436171_at

1436172_at
1436453_at
1436576_at
1436590_at
1436659_at
1436737_a_at
1436763_a_at
1436778_at
1436779_at
1436838_x_at
1436871_at
1436902_x_at
1436996_x_at
1437218_at
1437668_at
1437751_at
1437811_x_at
1437873_at
1438009_at
1438059_at
1438075_at
1438220_at
1438385_s_at
1438651_a_at
1438702_at
1438704_at
1438796_at
1438814_at
1438862_at
1438896_at
1438900_at
1438931_s_at
1439081_at

1439426_x_at
1439622_at
1439643_at
1439774_at
1439806_at
1439814_at
1439836_at
1439912_at
1440007_at
1440037_at
1440092_at
1440169_x_at
1440226_at
1440311_at
1440719_at
1441189_at
1441346_at
1441376_at
1441410_at
1441445_at
1441956_s_at
1441964_at
1442025_a_at
1442026_at
1442082_at
1442169_at
1442461_at
1442715_at
1442977_at
1443116_at
1443128_at
1443235_at
1443323_at

1443894_at
1443962_at
1443983_at
1444195_at
1444218_at
1444226_at
1444376_at
1444456_at
1444487_at
1444531_at
1444546_at
1444599_at
1445381_at
1445866_at
1445882_at
1446001_at
1446269_at
1446318_at
1446326_at
1446693_at
1447213_at
1447527_at
1447621_s_at
1448025_at
1448160_at
1448162_at
1448181_at
1448259_at
1448591_at
1448747_at
1448749_at
1448797_at
1449009_at

1449049_at
1449127_at
1449135_at
1449153_at
1449164_at
1449360_at
1449399_a_at
1449455_at
1449580_s_at
1449755_at
1449945_at
1449981_a_at
1450034_at
1450065_at
1450241_a_at
1450291_s_at
1450505_a_at
1450616_at
1450625_at
1450678_at
1450696_at
1450783_at
1450792_at
1450826_a_at
1450843_a_at
1450967_at
1451156_s_at
1451174_at
1451478_at
1451567_a_at
1451721_a_at
1451762_a_at
1451767_at

1451777_at
1451860_a_at
1451886_at
1451941_a_at
1452087_at
1452126_at
1452203_at
1452261_at
1452345_at
1452382_at
1452417_x_at
1452431_s_at
1452463_x_at
1452478_at
1452500_at
1452707_at
1452719_at
1452968_at
1453196_a_at
1453214_at
1453287_at
1453332_at
1453503_at
1453571_at
1453628_s_at
1453782_at
1454169_a_at
1454240_at
1454268_a_at
1454699_at
1454768_at
1454867_at
1455096_at

1455136_at
1455269_a_at
1455332_x_at
1455399_at
1455418_at
1455573_at
1455660_at
1455778_at
1455860_at
1456307_s_at
1456341_a_at
1456344_at
1456395_at
1456632_at
1456655_at
1456705_at
1456733_x_at
1456944_at
1457042_at
1457117_at
1457228_x_at
1457459_at
1457586_at
1457753_at
1457779_at
1458053_at
1458299_s_at
1458586_at
1458603_at
1459202_at
1459238_at
1459391_at
1459760_at

1459823_at
1460020_at
1460188_at
1460208_at
1460218_at
1460336_at
1460437_at
1460603_at
